# Milk exosomes-mediated miR-31-5p delivery accelerates diabetic wound healing through promoting angiogenesis

**DOI:** 10.1080/10717544.2021.2023699

**Published:** 2022-01-05

**Authors:** Chengqi Yan, Jing Chen, Cheng Wang, Meng Yuan, Yu Kang, Zihan Wu, Wenqing Li, Guolei Zhang, Hans-Günther Machens, Yuval Rinkevich, Zhenbing Chen, Xiaofan Yang, Xiang Xu

**Affiliations:** a Department of Hand Surgery, Union Hospital, Tongji Medical College, Huazhong University of Science and Technology, Wuhan, China; b Department of Hand and Foot Surgery, Huazhong University of Science and Technology, Union Shenzhen Hospital, Shenzhen, China; c Department of Plastic and Hand Surgery, Technical University of Munich, Munich, Germany; d Institute of Lung Biology and Disease, Helmholtz Zentrum München, Munich, Germany; e Institute of Regenerative Biology and Medicine, Helmholtz Zentrum München, Munich, Germany

**Keywords:** Milk-derived exosomes, miR-31-5p, drug delivery, diabetic wound, angiogenesis

## Abstract

The refractory diabetic wound has remained a worldwide challenge as one of the major health problems. The impaired angiogenesis phase during diabetic wound healing partly contributes to the pathological process. MicroRNA (miRNA) is an essential regulator of gene expression in crucial biological processes and is a promising nucleic acid drug in therapeutic fields of the diabetic wound. However, miRNA therapies have limitations due to lacking an effective delivery system. In the present study, we found a significant reduction of miR-31-5p expression in the full-thickness wounds of diabetic mice compared to normal mice. Further, miR-31-5p has been proven to promote the proliferation, migration, and angiogenesis of endothelial cells. Thus, we conceived the idea of exogenously supplementing miR-31-5p mimics to treat the diabetic wound. We used milk-derived exosomes as a novel system for miR-31-5p delivery and successfully encapsulated miR-31-5p mimics into milk exosomes through electroporation. Then, we proved that the miR-31-5p loaded in exosomes achieved higher cell uptake and was able to resist degradation. Moreover, our miRNA-exosomal formulation demonstrated dramatically improved endothelial cell functions *in vitro*, together with the promotion of angiogenesis and enhanced diabetic wound healing *in vivo*. Collectively, our data showed the feasibility of milk exosomes as a scalable, biocompatible, and cost-effective delivery system to enhance the bioavailability and efficacy of miRNAs.

## Introduction

Non-healing wound is one of the diabetic chronic complications, which not only causes large physiological and psychological pain to patients but also impose a tremendous burden on the entire economy and society (Boulton et al., 2005). Under normal conditions, the process of wound closure involves four overlapping and coordinated stages: hemostasis, inflammation, proliferation, and remodeling (Rodrigues et al., 2019). In patients with diabetic non-healing wounds, the wound healing process slows down or even stalls due to impairment and prolongation of the four stages (Davis et al., 2018; Zubair & Ahmad, 2019). Typically, the dysfunction of endothelial cells and microcirculation disorders occur very often in diabetic patients, which contributes to the impaired angiogenesis process, a process that happens at the beginning of the proliferation stage (Grennan, 2019).

MicroRNAs (miRNAs) are one class of short, evolutionarily conserved non-coding RNAs, which contain about 20–24 nucleotides (Ambros, 2001). A growing number of studies have reported that miRNAs have great potential as predictive, diagnostic, and even therapeutic biomarkers in many types of diseases, including diabetic wound healing (Ohtsuka et al., 2021; Zhang et al., 2017; Petkovic et al., 2020). For example, miR-126-3p promoted endothelial cell proliferation and migration, thus improving angiogenesis and wound healing in diabetic mice (Tao et al., 2017). Benefiting from its anti-inflammatory role, the application of miR-146a conjugated with cerium oxide nanoparticles enhanced diabetic wound healing (Zgheib et al., 2019). Despite the high potential of miRNA-based gene therapy, it has many obstacles that need to be overcome. Firstly, due to the negative charge of miRNAs, they are difficult to pass through cell membranes (Boca et al., 2020). In addition, miRNAs are unstable *in vitro* and are easy to degrade in the wound microenvironment (Meng et al., 2018). Therefore, the development of safe and efficient miRNA delivery systems is of great significance to optimize miRNA-based gene therapy.

Exosomes are one kind of cell-derived, nano‐sized membranous vesicles, which originate from the endosomal system and are 30–150 nm in diameter (EL Andaloussi et al., 2013). Accumulating studies have reported that exosomes and their cargos have a therapeutic effect in various diseases ranging from cancers, cardiovascular disease, to diabetic complications (Zheng et al., 2020; Chen et al., 2021; Sorop et al., 2021). Exosomes have attracted significant attention not only because of their therapeutic effect or their function as biomarkers but also because of their potential use for next‐generation drug delivery systems (Liang et al., 2021). Compared with the traditional gene carrier, the advantages of exosomes lie in the following aspects: (1) as an endogenous vesicle, exosomes have low immunogenicity; (2) their biocompatible structure makes them easy to enter cells, which enables gene delivery more efficient; (3) their robust membrane can protect therapeutic nucleic acids from degradation; (4) their ability to avoid phagocytosis and bypass engulfment by lysosomes makes them well-tolerated in body fluids; (5) their membrane can be artificially engineered for targeted delivery, which makes tissue-specific or cell-specific distribution possible (Pirisinu et al., 2020; Herrmann et al., 2021; Ullah et al., 2021; Yao et al., 2021).

However, the yield of exosomes from cell culture supernatant is so low that the application of exosomes for drug delivery is limited (Pirisinu et al., 2020). Therefore, the urgent task is to effectively obtain exosomes in mass quantities for further pharmaceutical applications. In 1973, Plantz et al. discovered the presence of exosomes in bovine milk, which is now considered a promising candidate in developing a new drug delivery system (Plantz et al., 1973; Munagala et al., 2016). Firstly, the quantity of exosomes isolated from bovine milk is relatively large, which is the biggest advantage that cannot be matched by any other sources of exosomes (Sedykh et al., 2020). In addition, milk-derived exosomes are highly resistant to harsh gastrointestinal tract environments and can be modified as an oral delivery vehicle (Aqil et al., 2016; Rani et al., 2017; Betker et al., 2019).

**Figure 1.**
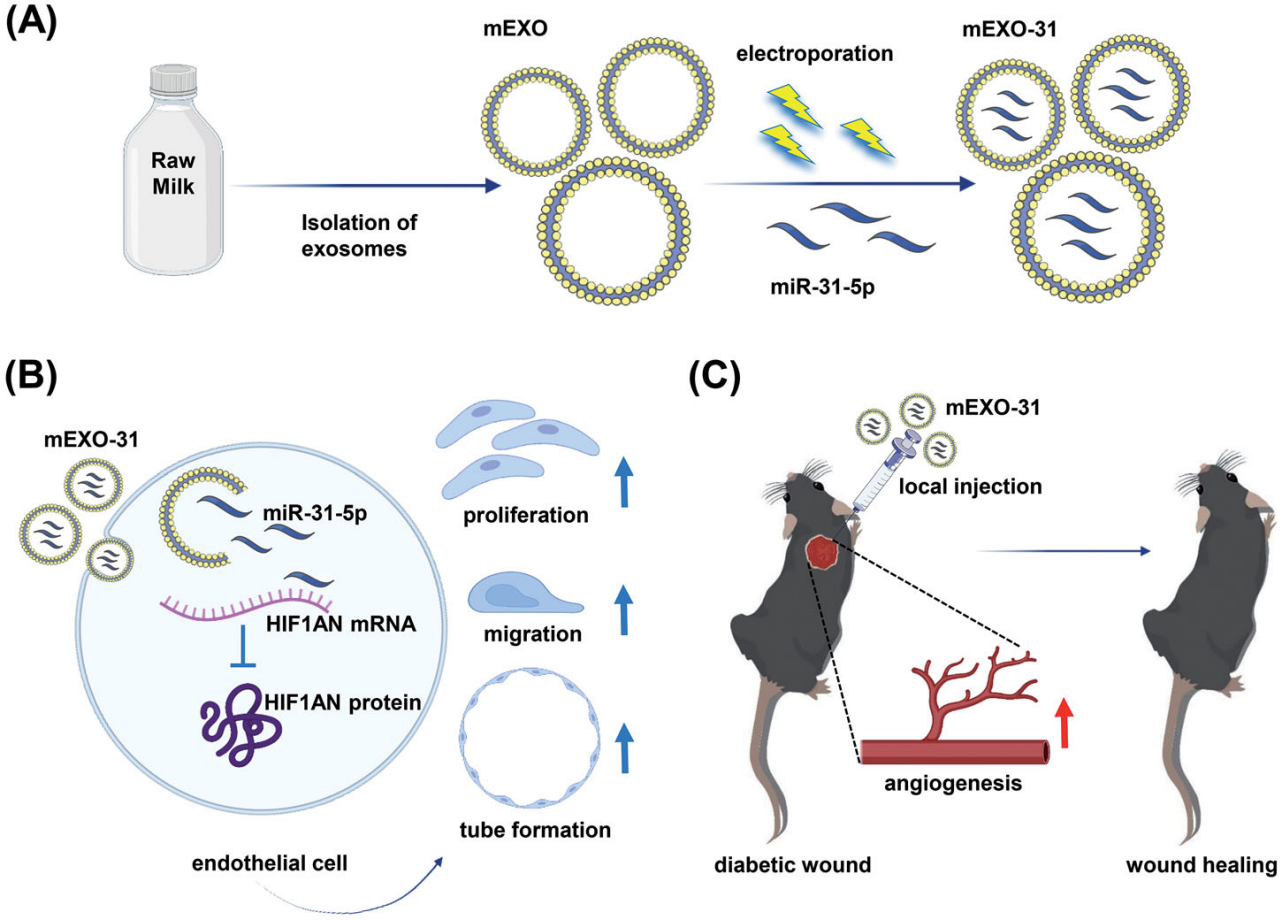
Schematic image. (A) Isolation of milk-derived exosomes (mEXO) and preparation of miR-31-5p-loaded exosomes (mEXO-31). (B) Action mechanism of mEXO-31 *in vitro*. (C) mEXO-31 treatment on mice model.

Here, we investigated the therapeutic efficacy of miR-31-5p encapsulated in milk exosomes in diabetic wound healing. We detected that miR-31-5p expression was reduced in skin wounds of diabetic mice compared with normal ones. Then, we isolated milk-derived exosomes (mEXO) by differential centrifugation and synthesized miR-31-5p-loaded exosomes (mEXO-31) by electroporation. We treated endothelial cells with mEXO-31 and found that miR-31-5p was upregulated in the recipient cells, while the target gene hypoxia-inducible factor 1 subunit alpha inhibitor (HIF1AN) was downregulated. Furthermore, the exosomes-coated miR-31-5p displayed potent pro-angiogenesis activity both *in vitro* and *in vivo* (Figure 1).

## Materials and methods

### Cell culture and transfection

Human umbilical vein endothelial cells (HUVECs) (#GDC166, CCTCC) were obtained from the China Center for Type Culture Collection (CCTCC, Wuhan, China) and grown in high-glucose Dulbecco’s modified Eagle’s medium (DMEM; Gibco, USA) supplemented with 10% fetal bovine serum (FBS; Gibco, USA), 100 U/ml of penicillin, and 100 μg/ml of streptomycin in a 5% CO_2_ humidified atmosphere at 37 °C. The miR-31-5p mimic, miRNA-31-5p inhibitor, and the relevant negative controls (mimic NC and inhibitor NC) were obtained from Ribobio company (Guangzhou, China). The sequences were presented in Table S1. HUVECs were transfected using riboFECT™CP Reagent according to the manufacturer’s instruction. The plasmids for overexpressing were purchased from Genomeditech Biotechnology (Shanghai, China). Cell transfection was performed with the NEOFECT™ DNA transfection reagent according to the manufacturer’s protocol. At 48 h after transfection, the cells were processed for *in vitro* assays.

### HUVECs proliferation, migration, and tube formation assay

HUVECs from different treated groups were grown in 96-well culture plates (4000 cells per well) for 12 h. After 2 h incubation with the EdU, the proliferation rates of HUVECs from each group were evaluated with Cell-Light EdU Apollo *In Vitro* Kit (Ribobio, Guangzhou, China). The 24-well Transwell Chamber (8.0 μm pore size, Corning, USA) was used for assessing the migration ability of HUVECs from different treated groups. Briefly, HUVECs (16,000 cells per well) suspended in DMEM without serum were added to the upper compartment and then incubated in a complete culture medium containing 10% FBS for 24 h. Then, HUVECs migrated to the bottom surface were stained with crystal violet staining (Solarbio, Beijing, China) and counted under microscopy. For tube formation assay, HUVECs from different treated groups were seeded in 96-well culture plates (25,000 cells per well) that had been coated with 70 μl Matrigel Basement Membrane Matrix (BD Biosciences, CA, USA). Tube formation was detected under microscopy at 6 h incubation. The total tube length of the endothelial tubes was measured by the angiogenesis plugin available from ImageJ.

### RT-PCR

MiRNA and total RNA were isolated using miRNA Purification Kit (CW0627S, CWBIO) and Ultrapure RNA Kit (CW0581M, CWBIO), respectively according to the manufacturer’s instruction. MiRNA and total RNA were reversely transcribed into cDNA using the Prime-Script^®^ RT reagent Kit (#RR037A, TaKaRa). Real-time PCR was performed on the StepOnePlus™ platform (Applied Biosystems, USA) using TB Green^®^ Premix Ex Taq™ II kit (#RR820A, TaKaRa). The primer sequences were presented in Tables S2, S3. The relative expression levels of targeted genes were calculated using the 2^−ΔΔCt^ method and normalized to β-tubulin or u6.

### Western blot

Total proteins were extracted by RIPA lysis buffer with proteinase inhibitor (Roche, Switzerland). An equal amount of total protein (20–40 μg) was separated by SDS-PAGE (Beyotime Biotechnology, Shanghai, China), transferred into the PVDF membrane (Millipore, USA), and then incubated overnight with primary antibodies specific for HIF1AN (Abcame, USA), β-tubulin (Proteintech, China), CD9 (Abcame, USA), CD81 (Abcame, USA), HSP70 (Abcame, USA), TSG101 (ABclonal, China). Then, the membrane was incubated with secondary antibodies (Aspen, China) for 1 h and exposed to X-ray film (UVP, USA).

### Dual-luciferase reporter gene assay

The 3′UTR of HIF1AN was inserted into pmirGLO-luciferase promoter vector. For the mutant assay, the binding motif TCTTGCC was replaced with ATCGAGG. HEK-293T cells were seeded and cultured on 24-well plates. Then, cells were co-transfected with the miRNAs (mimic NC, miR-31-5p mimic) and pmirGLO-luciferase promoter vector containing the WT or MUT 3′UTR of HIF1AN. After 48 h, cells were collected and luciferase was detected using Dual-Luciferase Reporter Assay (Promega Corporation, USA). The firefly luciferase signal was normalized to that of the renilla luciferase signal.

### Isolation of milk-derived exosomes (mEXO)

Exosomes were isolated by differential centrifugation from raw milk as described (Aqil et al., 2019; Tao et al., 2020). Briefly, milk was centrifuged at 13,000 × g at 4 °C for 30 min to remove fat globules, cells, and cell debris. The upper-fat layer and pellet in the bottom of the tube were discarded and the supernatant was collected. The supernatant was then centrifuged at 100,000 × g at 4 °C for 60 min to remove large particles and microvesicles. Then the supernatant was centrifuged at 145,000 × g for 90 min at 4 °C and the exosome pellet was collected and washed three times with PBS and filtered through a 0.22 μm filter membrane. Exosome suspension was stored at −80 °C until used.

### Preparation and characterization of miR-31-5p-loaded mEXO (mEXO-31)

Milk-derived exosomes (mEXO) were loaded with miR-31-5p mimics (mEXO-31) or mimic NC (mEXO-NC) by electroporation using the CUY21EDIT II (BEX, Japan) electroporation system. The electroporation mixture was prepared by mixing mEXO and miR-31-5p in a 1:1 (wt/wt) ratio in PBS, with the final concentration of mEXO in the mixture was 0.1 mg/ml. The mixture was transferred into ice-cold 0.4-cm cuvettes and electroporated for 10 cycles with a perforation voltage of 110 V, a perforation opening time of 6 ms, a perforation interval of 10 ms, a penetration voltage of 25 V, and a capacitance of 940 μF. Post-electroporation, the mixture was transferred into a new tube at 37 °C for 30 min. Then, the un-loaded mimics or mimic NC were removed through ultracentrifugation methods. The morphology and the size distribution of exosomes in each sample were detected by transmission electron microscope (TEM) and nanoparticle tracking analysis (NTA), and the protein level was quantified with Pierce BCA Protein Assay Kit (Aspen, China) as the manufacturer’s instructions. To measure the stability of the miR-31-5p loaded in exosomes, free miR-31-5p mimics mixed with mEXO and mEXO-31 were incubated at 37 °C for 5 days. The miR-31-5p levels remaining in each sample were detected through RT-PCR.

### Confocal microscopy

mEXOs were incubated with a red fluorescent dye (Dil, Biotium, USA) for 30 min, and then were centrifuged to remove contaminating dye and to obtain the labeled mEXOs. For internalization assay, HUVECs were seeded in 24-well culture plates (30,000 cells per well) for 12 h, and co-cultured with Dil-labeled mEXOs for 24 h. After incubation, cells were washed twice with PBS and fixed in 4% paraformaldehyde for 10 min; thereafter, the nucleic was stained with DAPI (Solarbio, Beijing, China) and the cytoskeleton was stained with FITC phalloidin (Yeasen Biotech Co., Shanghai, China) according to the manufacturer’s instructions. The mEXO uptake by cells was observed by using the laser scanning confocal microscope. For cell miR-31-5p uptake assay, FAM-labeled miR-31-5p mimics were obtained from Ribobio company (Guangzhou, China) and were encapsulated into Dil-labeled mEXOs through electroporation. HUVECs were seeded in 24-well culture plates (30,000 cells per well) for 12 h, and co-cultured with PBS, FAM-labeled miR-31-5p mimics, Dil-labeled mEXOs, and FAM-Dil-labeled mEXO-31, respectively for 24 h. After incubation, cells were washed twice with PBS and fixed in 4% paraformaldehyde for 10 min; thereafter, the nucleic was stained with DAPI (Solarbio, Beijing, China). The cell miR-31-5p uptake was observed by using the laser scanning confocal microscope.

### Diabetic wound model

All animal experiments were approved by the Animal Care Committee of Tongji Medical College. Eight-week-old male BALB/c mice were intraperitoneally injected with streptozotocin (STZ, 50 mg/kg) for 5 days, and after 2 weeks, the blood glucose was measured by a blood glucose monitor. Diabetic mice were successfully induced when the blood glucose was above 16.7 mM and maintained for another 4 weeks before full-thickness cutaneous wounds were formed. Before surgery, a total of 36 diabetic mice were anesthetized with pentobarbital sodium (Sigma–Aldrich) (1%, 50 mg/kg). After shaving and sterilization, a full-thickness excision wound at a diameter of 8 mm was performed on the back of all mice. Then the 36 mice were randomly divided into 6 groups: PBS (control) group; mimic NC group (2 nmol/wound); free miR-31-5p mimic group (2 nmol/wound); mEXO group (1.0 μg/μl); mEXO-NC group (1.0 μg/μl) and mEXO-31 group (1.0 μg/μl). Treatments were performed at day 0, 5, and 10 post-wounding. Digital photographs were taken at day 0, 5, 10, and 15, and the wound area was measured using the Image J software.

### Histological analysis

At day 15 post-wounding, the whole wound bed of each mouse was obtained for histological analysis. The wounds were fixed with 4% paraformaldehyde. After being dehydrated with a series of graded ethanol, the tissues were then embedded in paraffin and cut into 8 μm thick longitudinal sections before further staining. The rate of re-epithelialization was analysis by using hematoxylin and eosin (H&E) staining, and the degree of collagen accumulation was evaluated by Masson staining.

### Immunofluorescence analysis

To determine angiogenesis of wound beds, the sections were incubated with CD31, and α-SMA antibody (Abcam, USA) overnight at 4 °C. After being washed three times with PBS, the sections were incubated with a second antibody (Aspen, China) for 1 h at room temperature. The image was taken by a microscope and then analyzed by using ImageJ software.

### Statistics

All statistical analyses were performed using GraphPad Prism software (version 8.0.2, La Jolla, CA, USA). For the comparison of two groups, an unpaired Student’s *t*-test was applied. For group ≥3, a one-way analysis of variance (ANOVA) with Tukey *post-hoc* test was carried out. All data are presented as mean ± standard deviation (*SD*). Statistical significance was set at *p* < .05.

## Results

### Overexpression of miR-31-5p promoted endothelial cell proliferation, migration, and angiogenesis

**Figure 2.**
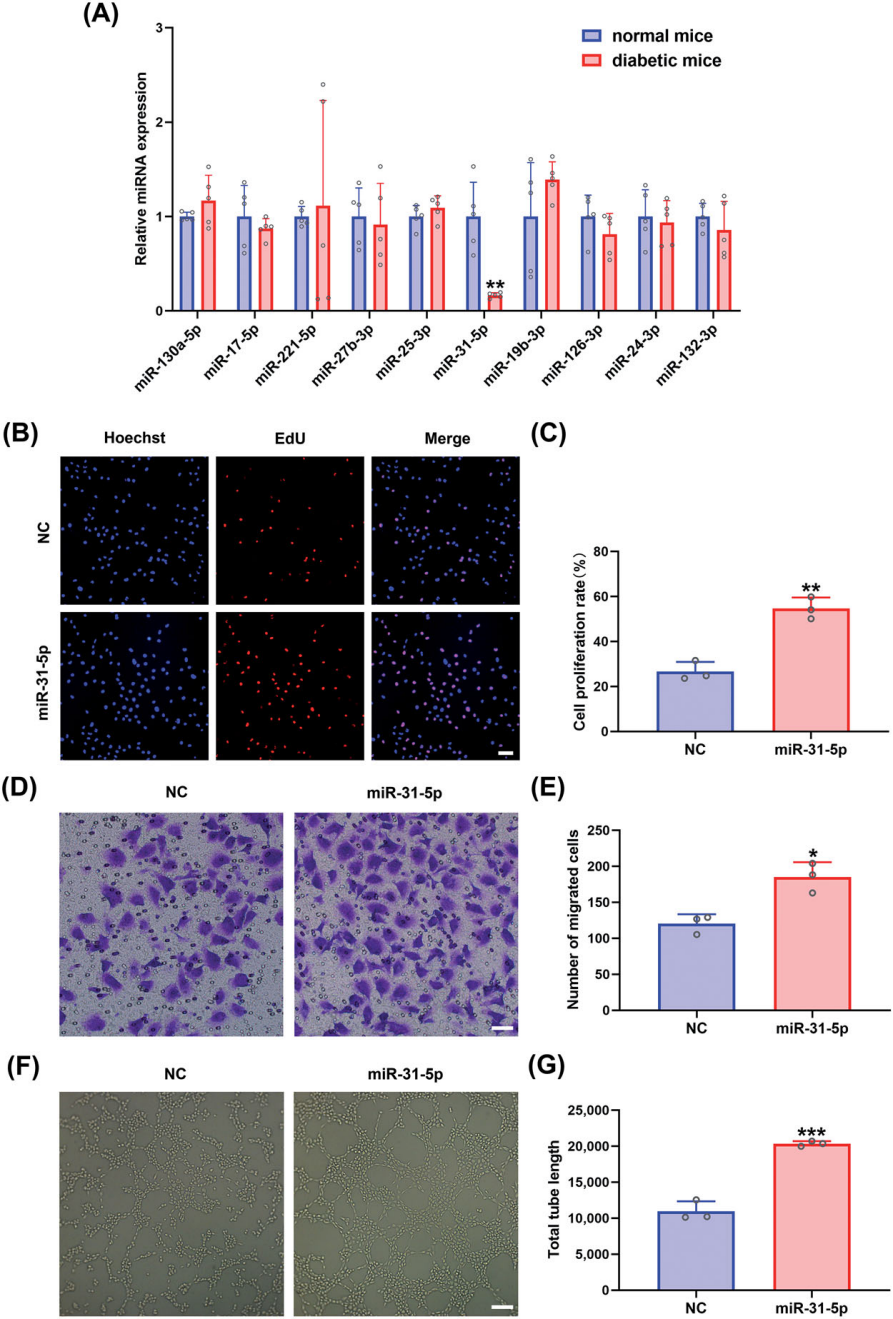
Overexpression of miR-31-5p promoted endothelial cell proliferation, migration, and angiogenesis. (A) Relative expression levels of miRNAs in wound tissue compared between normal mice and diabetic mice. *n* = 5, **p < .01 *vs.* normal mice. (B,C) EdU assay analysis of the proliferation rate of HUVECs treated with mimic-NC/miR-31-5p mimics. The proliferative cells and cellular nuclei were stained with red and blue colors. *n* = 3, **p < .01 *vs.* NC. Scale bar, 50 μm. (D,E) Images of migrated HUVECs in each group. *n* = 3, *p < .05 *vs.* NC. Scale bar, 50 μm. (F,G) Images of tube formation of HUVECs in each group. *n* = 3, ***p < .001 *vs.* NC. Scar bar, 100 μm. Data were presented as mean ± *SD*. Unpaired Student’s *t*-test was used.

To encapsulate a proangiogenic miRNA in milk exosomes to enhance diabetic wound healing, we compared the expression levels of ten miRNAs, including miR-130a-5p, miR-17-5p, miR-221-5p, miR-27b-3p, miR-25-3p, miR-31-5p, miR-19b-3p, miR-126-3p, miR-24-3p, and miR-132-3p, in full-thickness skin lesions between diabetic and normal mice to find the aberrantly downregulated miRNAs. Among five downregulated miRNAs in diabetes wounds (miR-17-5p, miR-27b-3p, miR-31-5p, miR-126-3p, and miR-132-3p), miR-31-5p showed statistically significant down regulation (Figure 2(A)). The significance of lower miR-31-5p was also tested in db/db T2DM murine model (Figure SF3(A)). To simulate the abnormally expressed miR-31-5p in diabetic wounds and to investigate the role of miR-31-5p, HUVECs were transfected with miR-31-5p inhibitors. We found that miR-31-5p inhibitors displayed an anti-proliferation, anti-migration, and anti-angiogenesis activity in HUVECs (Figure SF1). Thus, we hypothesized that the exogenous supplement of miR-31-5p could restore endothelial cell functions. After transfecting miR-31-5p mimics to HUVECs for 48 h, we conducted a series of assays below. Firstly, the proliferation of the cells was determined by EdU assays, and much more EdU-positive HUVECs were found when the cells were transfected with mimics (Figure 2(B,C)). Additionally, transwell assays showed that miR-31-5p significantly increased migration of HUVECs (Figure 2(D,E)). Lastly, *in vitro* tube formation assays were performed to assess the ability of miR-31-5p to promote endothelial cell angiogenesis. As we expected, there was a nearly 2-fold increase in total tube length, which indicated that miR-31-5p dramatically promoted angiogenesis (Figure 2(F,G)). In summary, these data suggest that miR-31-5p was down-regulated in diabetic mice, which may be one of the reasons for delayed wound healing, and the overexpression of miR-31-5p enhanced the HUVEC proliferation, migration, and angiogenesis *in vitro*.

### HIF1AN was a direct target of miR-31-5p

**Figure 3.**
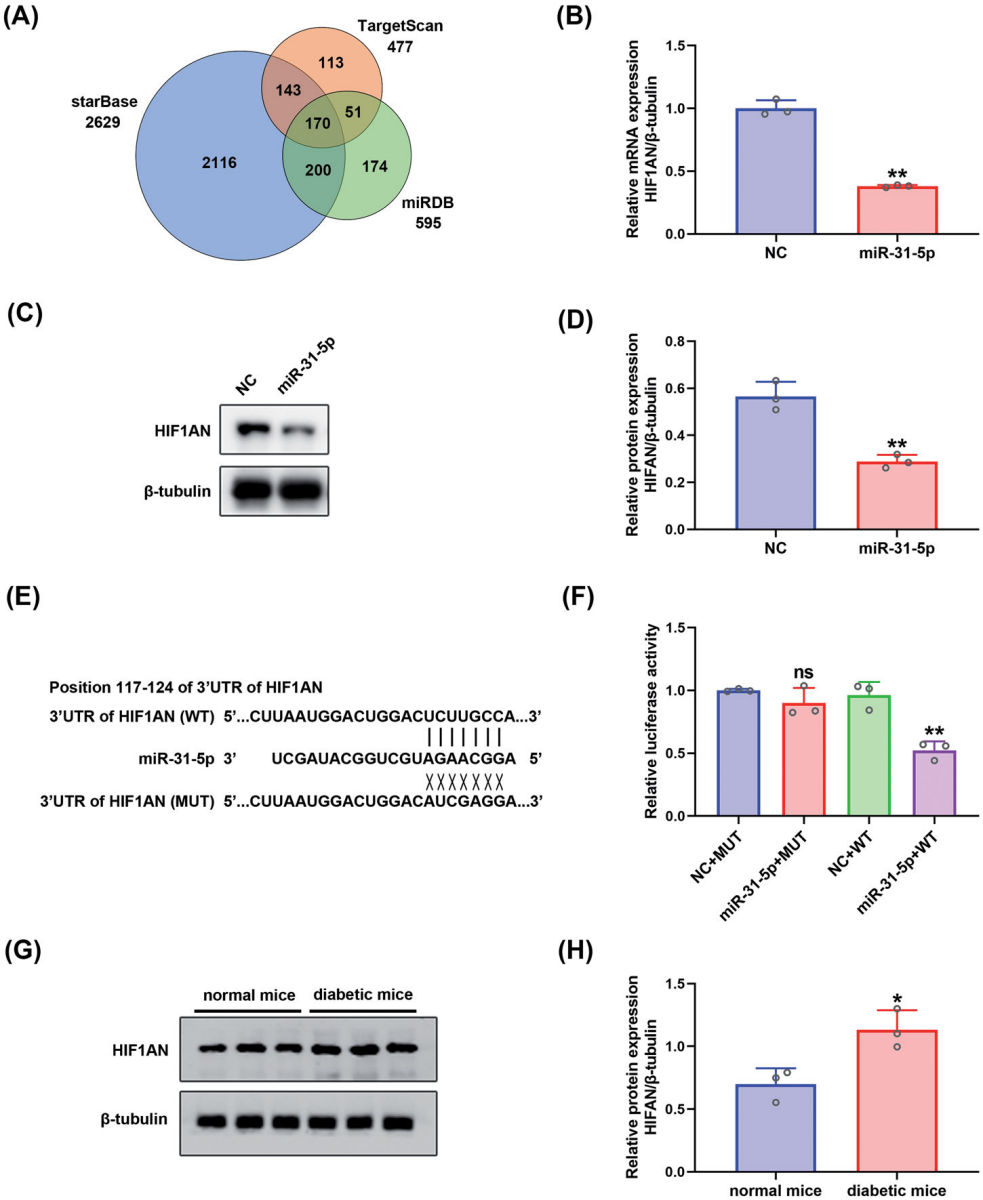
HIF1AN was a direct target of miR-31-5p. (A) Venn diagram depicting the number of potential targets of miR-31-5p predicted by three algorithms. (B) RT-PCR analysis of HIF1AN mRNA expression in HUVECs transfected with mimic-NC/miR-31-5p mimics. *n* = 3, **p < .01 *vs.* NC (C,D) Western blotting analysis of HIF1AN protein expression in HUVECs in each group. *n* = 3, **p < .01 *vs.* NC. (E) Schematic drawing of the putative binding sites or mutations of miR-31-5p in HIF1AN mRNA 3′UTR. (F) Luciferase activity of each group was detected at 48 h post-transfection. *n* = 3, ns no significant *vs.* NC + MUT; **p < .01 *vs.* NC + WT. (G,H) Western blotting analysis of HIF1AN protein expression in wound tissue from normal and diabetic mice. *n* = 3, *p < .05 *vs.* normal mice. Data were presented as mean ± *SD*. Unpaired Student’s *t*-test was used in (B,D,H). One-way ANOVA with Tukey *post-hoc* test was used in (F).

To seek out the downstream mechanisms governing the function of endothelial cells, three independent algorithms: starBase (Li et al., 2014), TargetScan (Agarwal et al., 2015), and miRDB (Chen & Wang, 2020) were used to predict the potential targets of miR-31-5p. 170 putative miR-31-5p targets were predicted by the three algorithms (Figure 3(A)). Then, we searched DAVID, a web-based functional annotation tool (Jiao et al., 2012), for functional annotations of the 170 genes. We found that HIF1AN, STARD13, and SPARC were three genes related to the negative regulation of angiogenesis. We selected HIF1AN for further study because the target score of HIF1AN was the highest among the three genes. The three algorithms revealed that HIF1AN was a conserved target of miR-31-5p with the pairing position of 117-124 in the 3′ untranslated region (UTR) of HIF1AN 3′UTR (Figure 3(E)). To further confirm that HIF1AN was a direct target of miR-31-5p, miR-31-5p mimics were transfected into HUVECs. We found that HIF1AN levels in miR-31-5p mimics transfected HUVECs decreased in both mRNA (Figure 3(B)) and protein (Figure 3(C,D)) levels. Then the luciferase reporters (pmirGLO-3′UTR of HIF1AN-WT and HIF1AN-MUT) were constructed and transfected into the 293 T cells. It was seen that overexpression of miR-31-5p apparently suppressed the luciferase activity of pmirGLO-HIF1AN-WT compared with empty vector control, while exerted no significant effects on the luciferase activity of pmirGLO-HIF1AN-MUT in 293 T cells (Figure 3(F)). In view of the association of HIF1AN with miR-31-5p, we also assessed the expression level of HIF1AN in wound tissue from diabetic mice via western blot. We then found an upregulation of HIF1AN in diabetic wounds compared to normal ones (Figure 3(G,H)), which presented the completely opposite tendency to miR-31-5p. The significance of higher HIF1AN was also tested in db/db T2DM murine model (Figures SF3(B,C)). Collectively, these results revealed that the expression of HIF1AN was upregulated in diabetic wounds and miR-31-5p could inhibit HIF1AN expression by binding to the conservative complementary sequence of the HIF1AN mRNA 3′UTR.

### miR-31-5p/HIF1AN axis regulated endothelial cell function

**Figure 4.**
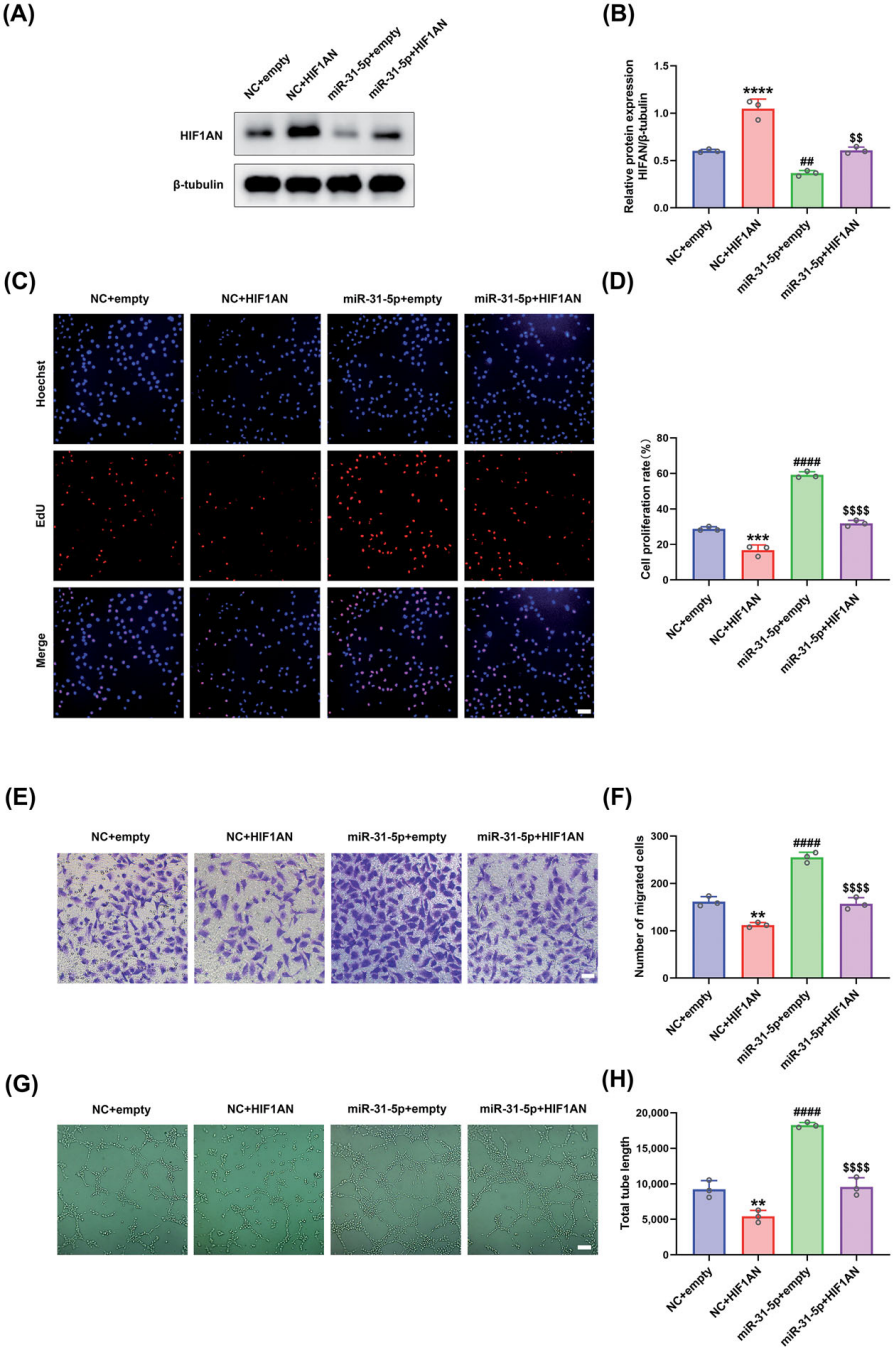
miR-31-5p/HIF1AN axis regulated endothelial cell function. (A,B) Western blotting analysis of HIF1AN protein expression in HUVECs in different treated groups. *n* = 3, ****p < .0001, ^##^*p* < .01 *vs.* NC + empty; ^$$^*p* < .01 *vs.* miR-31-5p + empty. (C,D) EdU assay analysis of the proliferation rate of HUVECs in each group. The proliferative cells and cellular nuclei were stained with red and blue colors. Scale bar, 50 μm. *n* = 3, ***p < .001, ^####^*p* < .0001 *vs.* NC + empty; *p* < .0001 *vs.* miR-31-5p + empty. (E,F) Images of migrated HUVECs in each group. Scar bar, 50 μm. *n* = 3, **p < .01, ^####^*p* < .0001 *vs.* NC + empty; *p* < .0001 *vs.* miR-31-5p + empty. (G,H) Images of tube formation of HUVECs in each group. Scar bar, 100 μm. *n* = 3, **p < .01, ^####^*p* < .0001 *vs.* NC + empty; *p* < .0001 *vs.* miR-31-5p + empty. Data were presented as mean ± *SD*. One-way ANOVA with Tukey *post-hoc* test was used.

We further studied the biological function of miR-31-5p/HIF1AN axis in HUVECs. Firstly, the protein levels of HIF1AN were detected in HUVECs under different treatments, showing that transfection of HIF1AN overexpressing plasmid achieved a higher level of HIF1AN than empty control plasmid, and rescued the reduced HIF1AN content induced by miR-31-5p mimics (Figure 4(A,B)). Then, EdU assays were performed to detect the proliferation activity of HUVECs. We demonstrated that the overexpression of HIF1AN exhibited a notable anti-proliferation effect and obviously counteracted miR-31-5p-improved HUVEC proliferation (Figure 4(C,D)). Moreover, a similar trend could be observed in the transwell assays detecting the migration activity of the cells (Figure 4(E,F)). We supposed that the overexpression of HIF1AN could inhibit the vascular formation of HUVECs, and the subsequent *in vitro* tube formation assays confirmed the hypothesis (Figure 4(G,H)). We also demonstrated that HIF1AN overexpression abolished the miR-31-5p mimic-induced promotion of angiogenesis (Figure 4(G,H)). In summary, our rescue experiments confirmed that miR-31-5p promoted endothelial cell functions through targeting HIF1AN.

### Preparation and characterization of mEXO-31

**Figure 5.**
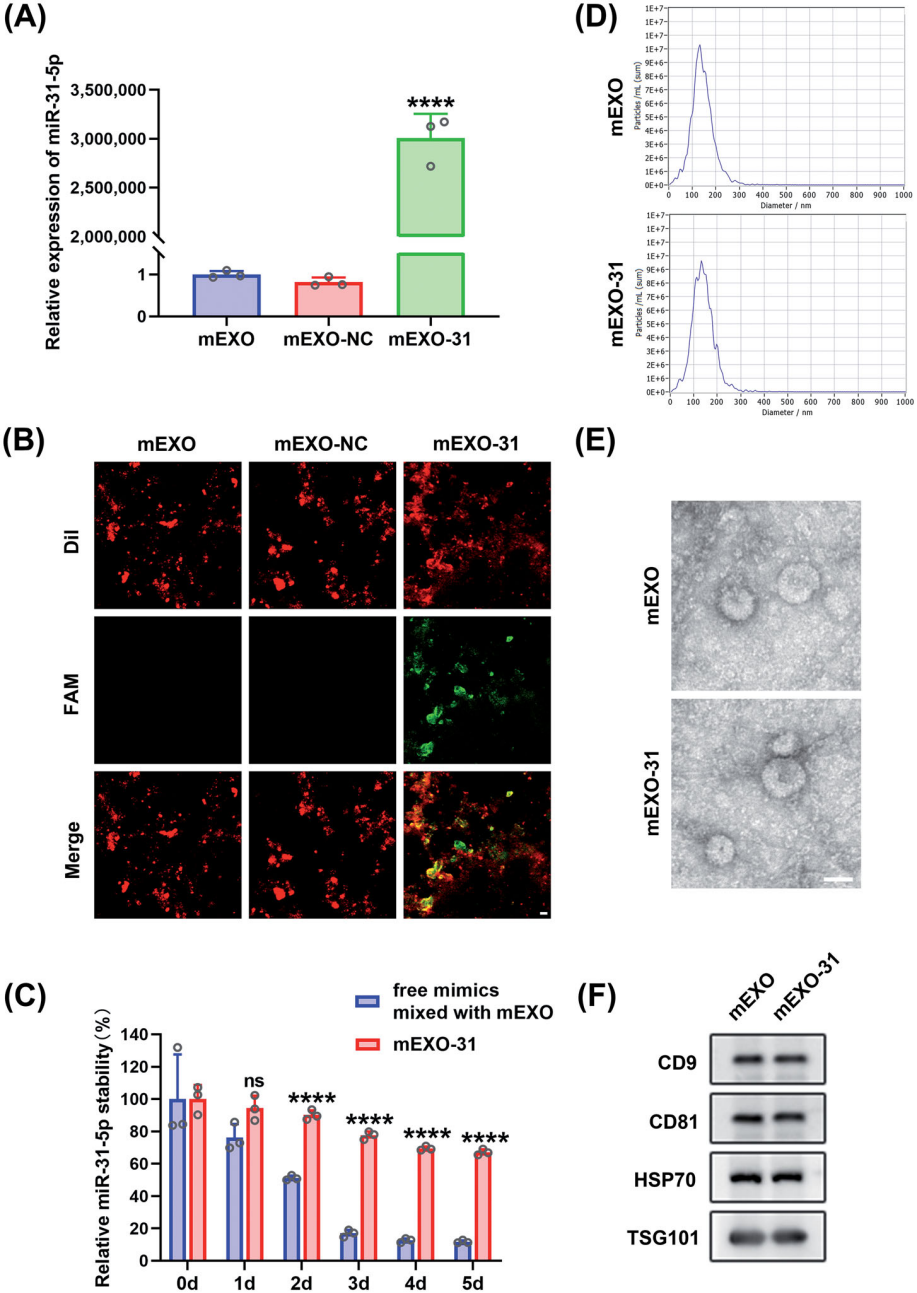
Preparation and characterization of mEXO-31. (A) RT-PCR analysis of relative miR-31-5p levels in mEXO, mEXO-NC, and mEXO-31. *n* = 3, ****p < .0001 *vs.* mEXO-NC. (B) Confocal images showed successful loading of miR-31-5p into mEXO. Red and green fluorescence represented mEXO and miR-31-5p mimics, respectively. Scale bar, 10 µm. (C) RT-PCR analysis of remaining miR-31-5p in each group. *n* = 3, ns no significant, ****p < .0001 *vs.* free mimics mixed with mEXO. (D) NTA identified the size distribution of mEXO and mEXO-31. (E) TEM identified the morphology of mEXO and mEXO-31. Scale bar, 50 µm. (F) Western blotting analysis of exosome specific markers including CD9, CD81, HSP70, and TSG101 of mEXO and mEXO-31. Data were presented as mean ± *SD*. One-way ANOVA with Tukey *post-hoc* test was used in (A). Unpaired Student’s *t*-test was used in (C).

To investigate whether milk-derived exosomes (mEXO) can function as an ideal cargo carrier to deliver miR-31-5p to endothelial cells and diabetic wounds, we isolated exosomes from raw milk using a differential centrifugation approach. We demonstrated the internalization and the biocompatibility of mEXO in HUVECs (Figure SF2). Then, we used electroporation approach to load mimic-NC and miR-31-5p mimics into mEXO (named as mEXO-NC and mEXO-31, respectively), followed by removing the unloaded NC and mimics through ultracentrifugation. To verify that miR-31-5p mimics were successfully loaded into mEXO, we detected the level of miR-31-5p in mEXO, mEXO-NC, and mEXO-31 by RT-PCR. The results showed that the miR-31-5p level was dramatically higher in mEXO-31 than in mEXO or mEXO-NC (Figure 5(A)). Moreover, confocal laser-scanning microscopy visually observed that FAM-labeled miR-31-5p mimics were successfully loaded into mEXO (Figure 5(B)).

The mean size of mEXO and mEXO-31 was 131.1 and 133.6 nm in diameter, respectively as measured by NTA (Figure 5(D)). Inwardly concave cuplike structure was found in the two exosomes as observed by TEM (Figure 5(E)). Western blotting analysis revealed that there was high expression of exosome-specific markers in the two exosomes including CD9, CD81, HSP70, TSG101 (Figure 5(F)). These data indicated that loading miRNA by electroporation into mEXO had almost no influence on the particle size, morphology, or surface markers of the mEXO.

Further, we explored the extent to which the mEXO protected the miRNA. Free miR-31-5p mimics mixed with mEXO and mEXO-31 were exposed to 37 °C for 5 days, and then the miR-31-5p level was quantified from each sample. The results showed that free miR-31-5p mimics almost totally degraded at day 5, on the contrary, more than 60% of the miR-31-5p loaded in mEXO remained stable (Figure 5(C)), indicating that mEXO had the ability to protect miR-31-5p mimics from degradation.

### mEXO-31 delivered miR-31-5p into cells more efficiently

**Figure 6.**
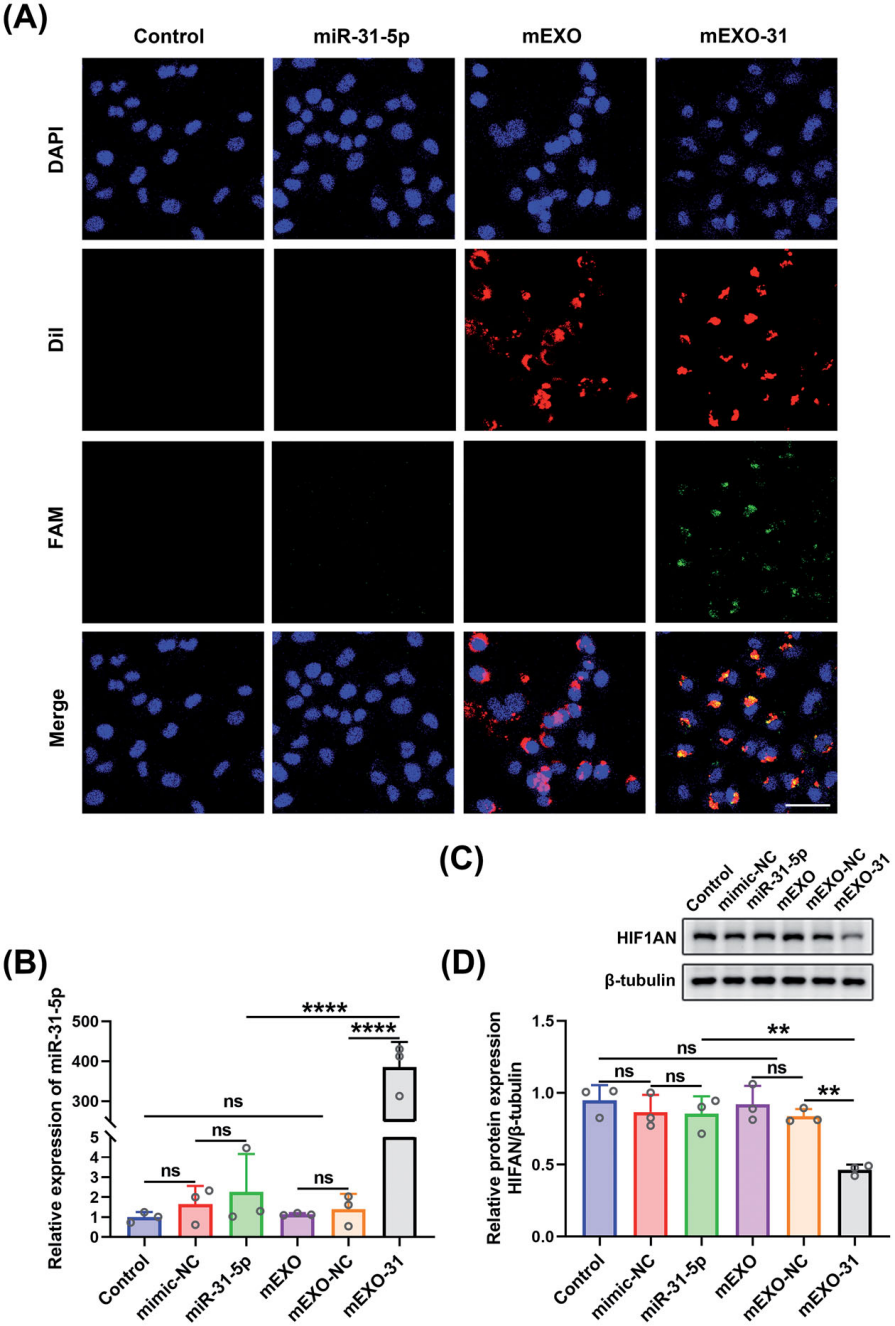
mEXO-31 delivered miR-31-5p into cells more efficiently. (A) Confocal images showed the delivery of miR-31-5p into HUVECs in each group. Blue, red, and green fluorescence represented the cellular nuclei, mEXO, and miR-31-5p, respectively. Scale bar, 50 µm. (B) RT-PCR analysis of relative miR-31-5p levels in HUVECs in different treated groups. (C,D) Western blotting analysis of HIF1AN protein expression in HUVECs in different treated groups. *n* = 3, ns: no significant, **p < .01, ****p < .0001. Data were presented as mean ± *SD*. One-way ANOVA with Tukey *post-hoc* test was used.

For the sake of detecting the transfection efficiency of mEXO-31, we transfected HUVECs with PBS, free miR-31-5p mimics, mEXO, and mEXO-31, respectively, and determined the efficiency using confocal laser-scanning microscopy. As shown in Figure 6(A), almost all HUVECs were successfully transfected with miR-31-5p when the cells were treated with mEXO-31, while nearly no miR-31-5p was detected in the cells when treated with free mimics. We then respectively treated HUVECs with PBS, free mimic-NC, free miR-31-5p mimics, mEXO, mEXO-NC, and mEXO-31. RT-PCR was used to detect the expression level of miR-31-5p in HUVECs, showing that there was a remarkable increase of miR-31-5p level in mEXO-31 group than in mEXO-NC and free mimics group (Figure 6(B)). In addition, the protein level of HIF1AN was significantly downregulated in HUVECs treated with mEXO-31, while the level of HIF1AN remained unchanged in other groups (Figure 6(C,D)). These results collectively indicated that naked free miRNA was difficult to be taken up by cells, and the dilemma of miRNA-based therapeutics could be broken by the milk exosome delivery system. More than that, the biological function of miR-31-5p encapsulated in mEXO was preserved, and it could reduce the expression of the target gene HIF1AN.

### mEXO-31 promoted endothelial cell proliferation, migration, and angiogenesis in vitro

**Figure 7.**
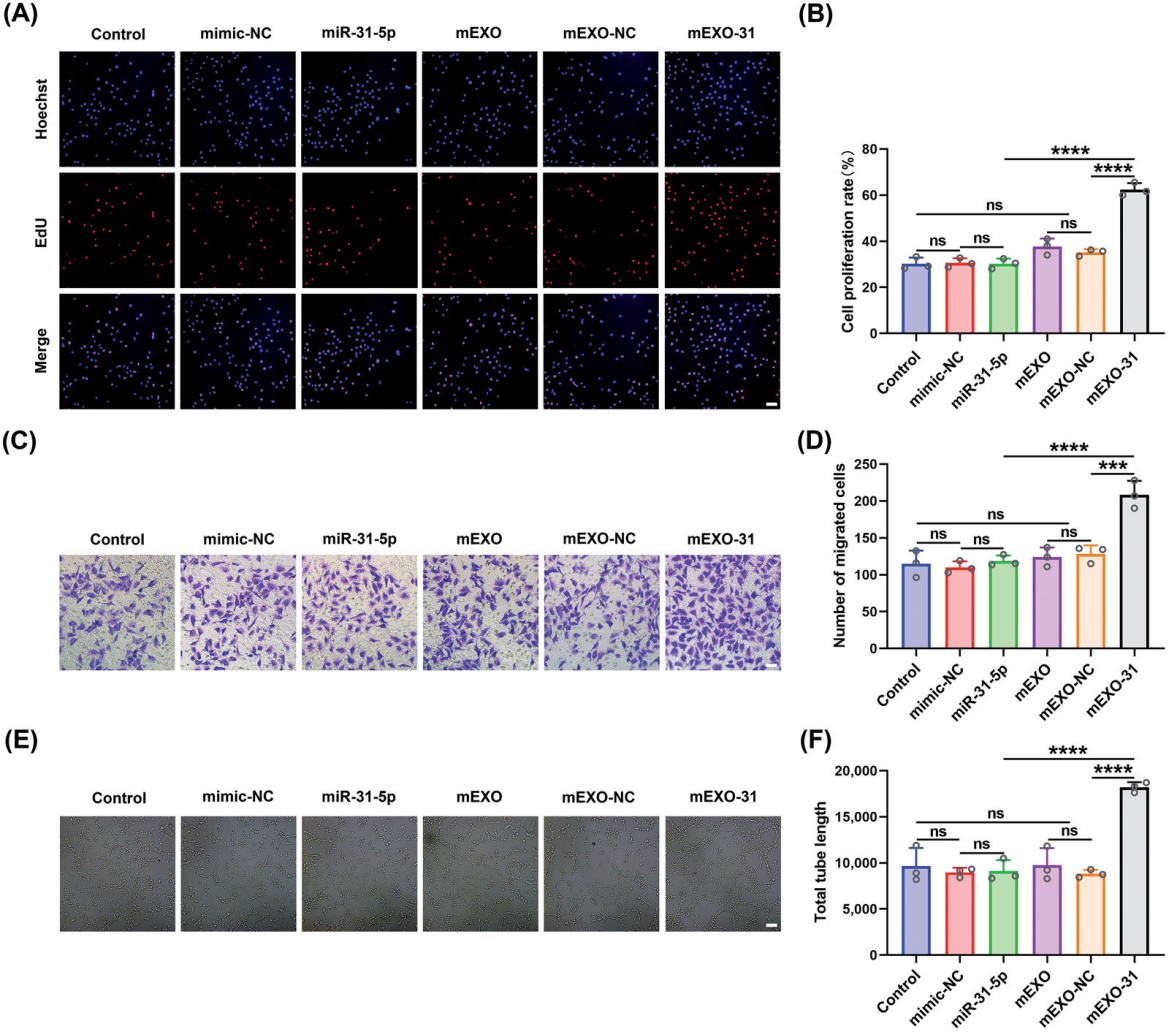
mEXO-31 promoted endothelial cell proliferation, migration, and angiogenesis *in vitro*. (A,B) EdU assay analysis of the proliferation rate of HUVECs in different treated groups The proliferative cells and cellular nuclei were stained with red and blue colors. Scale bar, 50 μm. (C,D) Images of migrated HUVECs in each group. Scar bar, 50 μm. (E,F) Images of tube formation of HUVECs in each group. Scar bar, 100 μm. *n* = 3, ns: no significant, ***p < .001, ****p < .0001. Data were presented as mean ± *SD*. One-way ANOVA with Tukey *post-hoc* test was used.

To further investigate the functional effects of mEXO-31 on HUVECs, we treated HUVECs with PBS, free mimic-NC, free miR-31-5p mimics, mEXO, mEXO-NC, and mEXO-31, respectively. Then, we evaluated the cell behavior by using EdU assays, transwell assays, and *in vitro* tube formation assays. Firstly, EdU assays were performed to assess the proliferation ability of endothelial cells. Consistent with our expectations, mEXO-31 treatment greatly promoted cell proliferation: the rate of EdU-positive HUVECs was increased more than 2-fold in mEXO-31 group compared with other groups (Figure 7(A,B)). Next, we evaluated the migration ability of HUVECs by conducting transwell assays. After being treated for 2 days, the cells were seeded in a transwell system. As the results showed, significant differences were observed between the mEXO-31 group and other groups, with migration increasing about 1.5-fold when HUVECs were treated with mEXO-31 (Figure 7(C,D)). Furthermore, the angiogenic tube-forming ability of endothelial cells was detected by vascular network formation assays in matrigel. The results revealed that stimulation of HUVECs with mEXO-31 notably promoted cell network tube formation *in vitro*, exhibiting a nearly 2-fold increase in total tube length in mEXO-31 group than in other groups (Figure 7(E,F)). The above evidence strongly suggested that neither mEXO nor free miR-31-5p mimics could promote endothelial cell functions, while delivery miR-31-5p by mEXO notably enhanced the bioavailability of miR-31-5p, thus improving the proliferation, migration, and tube formation of HUVECs.

### mEXO-31 accelerated diabetic wound healing in vivo

**Figure 8.**
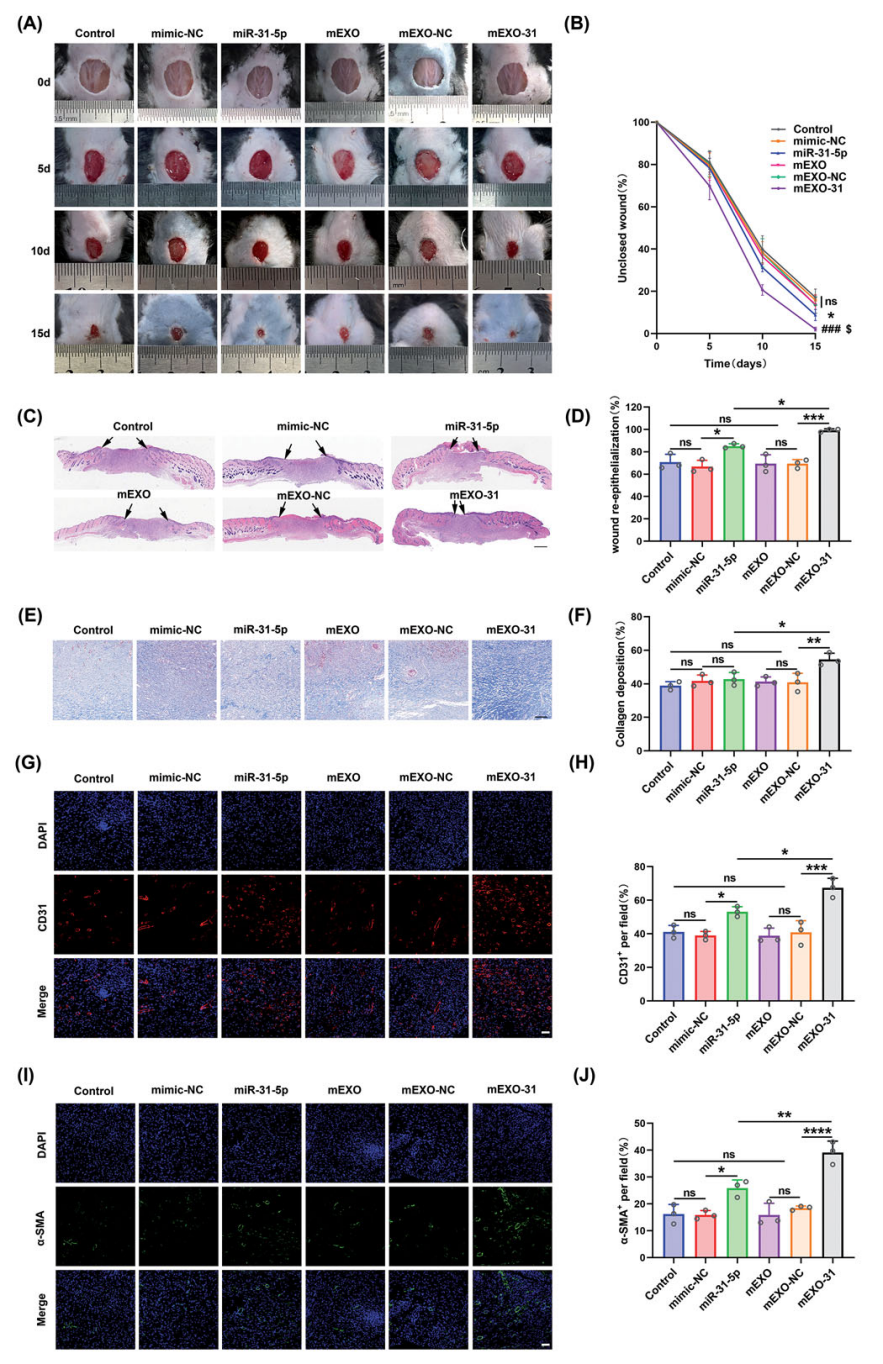
mEXO-31 accelerated diabetic wound healing *in vivo*. (A,B) Representative images of wound closure in a wound model at the dorsum of diabetic mice at day 0, 5, 10, and 15 post-wounding. *n* = 5, ns: no significant, *p < .05 *vs.* mimic-NC; ^###^*p* < .001 *vs.* mEXO-NC; ^$^*p* < .05 *vs.* miR-31-5p. (C,D) H&E staining analysis of wound sections at day 15 post-wounding. The single-headed arrows indicate the un-epithelialized areas. Scar bar, 500 μm. (E,F) Masson staining evaluated the collagen deposition at day 15 post-wounding. Scar bar, 100 μm. (G–J) Immunofluorescence staining of cellular nuclei (DAPI, blue), CD31 (red), and α-SMA (green) of wound bed at day 15 post-wounding. Scar bar, 50 μm. *n* = 3, ns: no significant, *p < .05, **p < .01, ***p < .001, ****p < .0001. Data were presented as mean ± *SD*. One-way ANOVA with Tukey *post-hoc* test was used.

To explore the potential of mEXO-31 for clinical treatment, we established a full-thickness wound model at the dorsum of diabetic mice, and used PBS, free mimic-NC, free miR-31-5p mimics, mEXO, mEXO-NC, and mEXO-31 to treat the mice. As we expected, the unclosed wound rate in mice treated with mEXO-31 was the smallest in these different treated groups at days 5, 10, and 15 post-wounding (Figure 8(A,B)). Interestingly, in the free miR-31-5p mimics treated diabetic wounds, the unclosed wound rate was smaller than that in control, NC, mEXO, and mEXO-NC groups (Figure 8(A,B)). This phenomenon may be attributed to our added treatment in each group at days 5 and 10 post-wounding. However, the therapeutic effect of free miR-31-5p mimics was far from satisfactory compared to mEXO-31. Next, histological analysis was performed to investigate the wound repair efficiency in different treatment groups at day 15 post-operation. H&E staining analysis showed that mEXO-31 treated group exhibited the highest re-epithelialization rate among other groups (Figure 8(C,D)). We also observed an enhanced re-epithelialization rate in the free mimics group (Figure 8(C,D)). In addition, the Masson staining analysis demonstrated that the collagen deposition in mEXO-31 group was better than that of other groups (Figure 8(E,F)). We observed no difference in collagen deposition between the mimics group and control, NC, mEXO, or mEXO-NC group (Figure 8(E,F)). CD31 and α-SMA are two indicators of blood vessels and were used to evaluate the angiogenesis in the diabetic wound bed. As shown in Figure 8(G–J), very few blood vessels were observed in the wound tissues of the control, NC, mEXO, and mEXO-NC group, while the treatment of free miR-31-5p mimics improved vascular network formation. Notably, the highest vascular density was achieved in mEXO-31 group. These data indicated that mEXO-31 significantly accelerated diabetic wound healing by promoting wound re-epithelialization, collagen deposition, and angiogenesis.

## Discussion

In the present study, miR-31-5p was selected from ten miRNA candidates to encapsulate in milk exosomes to accelerate diabetic wound healing. Comprehensive literature demonstrated that these ten miRNAs have been reported to be involved in the process of angiogenesis in diabetic complications and other diseases (Chen & Gorski, 2008; Rawal et al., 2017; Zhang et al., 2017). For instance, bone marrow-derived angiogenic cells (BMACs) exhibit impaired function in diabetes. While overexpression of miR-27b-3p could alleviate impaired BMAC function by improving cell proliferation, tube formation, adhesion, and delaying apoptosis. Moreover, cell therapy of BMACs overexpressing miR-27b-3p on diabetic wounds augmented wound perfusion and capillary formation (Wang et al., 2014). Among the 10 miRNAs, miR-31-5p was the only miRNA showing statistically significant downregulation in skin wounds of diabetic mice compared with normal ones.

Several studies have also investigated the aberrantly expressed miR-31-5p in diabetic complications. For example, the level of miR-31-5p was found to be downregulated in skin wounds of diabetic rats (Liu et al., 2015). Serum miR-31-5p level was reduced in T2D patients with diabetic nephropathy (Rovira-Llopis et al., 2018). During the wound healing process, previous studies have indicated that miR-31-5p played a crucial role in promoting keratinocyte function (Li et al., 2015; Shi et al., 2018). One study found that miR-31-5p was up-regulated in wound edge keratinocytes, and it promoted the proliferation and migration of keratinocytes by suppressing epithelial membrane protein 1 (EMP-1) (Li et al., 2015). Another study attributed the improved keratinocyte function to the miR-31-5p-mediated activation of the Ras/mitogen-activated protein kinase signaling (Shi et al., 2018). We showed here that miR-31-5p can also accelerate diabetic wound healing by improving endothelial cell function and promoting angiogenesis.

Consistent with our findings, miR-31-5p has been shown to be a proangiogenic miRNA in cancer and other diseases (Wu et al., 2011; Wang et al., 2014; Wong et al., 2015). It has been reported that miR-31-5p could induce endothelial motility and angiogenesis by targeting FAT atypical cadherin 4 (FAT4) (Wu et al., 2011). Similarly, overexpression of miR-31-5p or knockdown of FAT4 promoted endothelial progenitor cell (EPC) migration and tube formation in coronary artery disease (CAD) (Wang et al., 2014). The adhesion molecule E-selectin has been proven to be another target gene of miR-31-5p, and inhibition of E-selectin by miR-31-5p benefited HUVEC migration and angiogenesis (Suárez et al., 2010). In the current study, we demonstrated that miR-31-5p can positively regulate the proliferation, migration, and tube formation in HUVECs by targeting HIF1AN.

HIF1AN is an asparaginyl hydroxylase enzyme and it has been proven to be a negative regulator of HIF-1 transactivation domain function (Mahon et al., 2001; Lando et al., 2002). The active form of HIF-1 is important to induce a beneficial proangiogenic response in patients with diabetic ulcers or non-healing wounds (Rey & Semenza, 2010; Hong et al., 2014). Several studies have observed that targeting HIF1AN contributed to improved angiogenesis in endothelial cells (Huang et al., 2011; Umezu et al., 2014). We validated that HIF1AN is a direct target of miR-31-5p in HUVECs through bioinformatics analysis, PCR analysis, western blotting, and luciferase reporter assay. This regulatory relationship between miR-31-5p and HIF1AN agrees with previous studies (Liu et al., 2010; Peng et al., 2012; Cheung et al., 2014; Hu et al., 2015; Zhu et al., 2019). Then, we found that the expression of HIF1AN was upregulated in diabetic wounds. Interestingly, a marked increase of HIF1AN expression was also observed in corneal epithelium from patients with diabetic keratopathies (Peng et al., 2012). The function of HIF1AN and the relationship between miR-31-5p and HIF1AN was further clarified in our rescue experiments, where the overexpression of HIF1AN suppressed cell function and counteracted the promoting effects of miR-31-5p in HUVECs.

In view of the dramatically downregulated miR-31-5p in diabetic wounds and its essential role in angiogenesis, we wondered whether miR-31-5p replacement technology could be used to restore its expression and promote diabetic wound healing. As mentioned above, miRNAs are extensively researched for their gene regulation activity, and miRNA-based therapeutics present an attractive proposition for the development of genetic therapeutics during wound healing (Chouhan et al., 2019; Nie et al., 2020). However, the utilization of miRNAs as a solution for wound healing often meets with several limitations. Firstly, naked free miRNAs are susceptible to degradation in the external environment. Not only that, the high molecular weight of miRNAs and the repulsion between negatively charged miRNAs and cell membrane hinders the successful cell uptake. As shown in our research, free miR-31-5p mimics placed in the 37 °C incubators almost totally degraded in 5 days. And nearly no miR-31-5p was detected to be taken up by cells when they were treated with naked free mimics. Hence, strong efforts must be undertaken to exploit a delivery medium to carry the miRNAs so that the enhanced retention and permeability of miRNAs in the wound environment can be achieved.

Over the last decade, several synthetic materials-based delivery systems have been reported to carry miRNAs, including polymeric nanoparticles, liposomes, peptides, and synthetic dendrimers (Liu et al., 2017; Ben-Shushan et al., 2014; Zhou et al., 2016). Despite great achievements, the use of synthetic materials-based delivery systems suffers from their poor stability, low loading efficiency, lack of scalability, and difficulty in synthesizing them in sufficient quantities (Feng & Mumper, 2013; Lim et al., 2013). Moreover, immune response, non-specific targeting, and toxicity concerns with the long-term use limit their clinical application (Sadasivam et al., 2013). Thus, we emphasized exosomes as an ideal miRNAs delivery system for the next generation of genetic therapeutics. Compared to traditional synthetic nanoparticles, a natural exosomal drug delivery system is biocompatible, safe, and efficacious (Ha et al., 2016; Rani & Ritter, 2016). In recent years, researchers have established several miRNA delivery systems using exosomes from numerous cell types (Liang et al., 2018; Ma et al., 2018; Jeong et al., 2020; Kim et al., 2020; Liang et al., 2020). For example, human embryonic kidney 293 T (HEK293T) cell-derived exosomes were used as delivery vehicles for miR-497, and the miR-497-loaded exosomes exhibited anti-tumor and anti-angiogenic effects on non-small cell lung cancer (Jeong et al., 2020).

Although some promising results have been reported to isolate exosomes from cells for delivering hydrophilic and hydrophobic drugs, the workload required to scale up the production of these cells combined with low exosome yield hamper the delivery system for clinical application (Smyth et al., 2015). In view of the current limitations of cell-derived exosomes and scalability issues, researchers have recognized milk as a potential source of exosomes, since large quantities of exosomes can be harvested from ordinary milk on an industrial scale. Arntz et al isolated exosomes from commercial semi-skimmed milk with a reported yield of 200 mg protein per liter of milk (Arntz et al., 2015). Another two researches collected exosomes from raw milk with a higher yield, about 300 mg exosomes per liter of milk (Munagala et al., 2016; Tao et al., 2020). Our exosome yield was around 200 mg per liter of raw milk. Thus, compared to very low yields of exosomes isolated from the medium of cultured cells, about 0.5–2.0 mg exosomal protein one liter of cell culture supernatant, the exosome yield from milk is over 200-fold higher, indicating that milk is a scalable source of exosomes for mass production (Sheng et al., 2011; Munagala et al., 2016; Faruqu et al., 2018).

Milk exosomes can be potentially loaded with gene therapeutic drugs (Aqil et al., 2019; Matsuda et al., 2020; Tao et al., 2020; Del Pozo-Acebo et al., 2021). Recent studies have employed passive strategies, such as incubation and active cargo-loading methods, such as sonication, extrusion, freeze and thaw cycles, electroporation, and saponin treatment to load drugs into milk exosomes (Luan et al., 2017; Adriano et al., 2021; Kandimalla et al., 2021). Here, we demonstrated successful loading of miR-31-5p mimics into milk exosomes by electroporation. Accumulating evidence have verified that the robust membrane of exosomes can protect therapeutic nucleic acid drugs from degradation (Aqil et al., 2019; Jeong et al., 2020; Tao et al., 2020; Del Pozo-Acebo et al., 2021). For instance, one study found that almost all naked free siRNA molecules were degraded after incubating at 37 °C for 8 h, while 67.1 ± 8.3% of the exosome-encapsulated siRNA retained the integrity after 24 h (Tao et al., 2020). Our present study showed that free miR-31-5p almost totally degraded incubated at 37 °C in 5 days, on the contrary, more than 60% of the miR-31-5p loaded in milk exosomes remained stable. In agreement with other studies, the effectiveness of our miRNA loaded exosomes has been proven *in vitro* and *in vivo*, which identified milk-derived exosomes as a biocompatible, safe, and cost-effective delivery system to enhance bioavailability and efficacy of drugs (Aqil et al., 2016; Munagala et al., 2016; Munagala et al., 2017; Betker et al., 2019; Del Pozo-Acebo et al., 2021; Luo et al., 2021).

## Conclusion

In conclusion, we developed a new strategy for accelerating diabetic wound healing by loading miR-31-5p into milk-derived exosomes. We showed here that raw milk can serve as a biocompatible and economical source for harvesting large quantities of exosomes, and that milk exosomes have tremendous potential as a miRNA delivery system, which can enhance the stability and cell uptake of miRNA. Our exosomal formulation of miR-31-5p significantly improved endothelial cell functions and enhanced the healing process of the diabetic wound by downregulating the expression of HIF1AN. Such effects of miR-31-5p exosomes shed light on its role in treating a diabetic wound, and we foresee a great potential of milk exosome utilization in nucleic acid drug delivery.

## Supplementary Material

Supplemental Material

## Data Availability

The authors confirm that the data supporting the findings of this study are available within the article and its supplementary materials.

## References

[CIT0001] Adriano B, Cotto NM, Chauhan N, et al. (2021). Milk exosomes: nature's abundant nanoplatform for theranostic applications. Bioact Mater 6:2479–90. 33553829 10.1016/j.bioactmat.2021.01.009 PMC7856328

[CIT0002] Agarwal V, Bell GW, Nam JW, et al. (2015). Predicting effective microRNA target sites in mammalian mRNAs. Elife 4:e05005. 10.7554/eLife.05005 PMC4532895 26267216

[CIT0003] Agrawal AK, Aqil F, Jeyabalan J, et al. (2017). Milk-derived exosomes for oral delivery of paclitaxel. Nanomedicine 13:1627–36. 28300659 10.1016/j.nano.2017.03.001

[CIT0004] Al-Kafaji G, Al-Mahroos G, Al-Muhtaresh HA, et al. (2016). Decreased expression of circulating microRNA-126 in patients with type 2 diabetic nephropathy: a potential blood-based biomarker. Exp Ther Med 12:815–22. 27446281 10.3892/etm.2016.3395 PMC4950785

[CIT0005] Ambros V. (2001). MicroRNAs: tiny regulators with great potential. Cell 107:823–6. 11779458 10.1016/s0092-8674(01)00616-x

[CIT0006] Aqil F, Kausar H, Agrawal AK, et al. (2016). Exosomal formulation enhances therapeutic response of celastrol against lung cancer. Exp Mol Pathol 101:12–21. 27235383 10.1016/j.yexmp.2016.05.013

[CIT0007] Aqil F, Munagala R, Jeyabalan J, et al. (2019). Milk exosomes – natural nanoparticles for siRNA delivery. Cancer Lett 449:186–95. 30771430 10.1016/j.canlet.2019.02.011

[CIT0008] Arntz OJ, Pieters BCH, Oliveira MC, et al. (2015). Oral administration of bovine milk derived extracellular vesicles attenuates arthritis in two mouse models. Mol Nutr Food Res 59:1701–12. 26047123 10.1002/mnfr.201500222

[CIT0009] Ben-Shushan D, Markovsky E, Gibori H, et al. (2014). Overcoming obstacles in microRNA delivery towards improved cancer therapy. Drug Deliv Transl Res 4:38–49. 25786616 10.1007/s13346-013-0160-0

[CIT0010] Betker JL, Angle BM, Graner MW, et al. (2019). The potential of exosomes from cow milk for oral delivery. J Pharm Sci 108:1496–505. 30468828 10.1016/j.xphs.2018.11.022 PMC6788294

[CIT0011] Boca S, Gulei D, Zimta A, et al. (2020). Nanoscale delivery systems for microRNAs in cancer therapy. Cell Mol Life Sci 77:1059–86. 31637450 10.1007/s00018-019-03317-9 PMC11105078

[CIT0012] Boulton AJM, Vileikyte L, Ragnarson-Tennvall G, et al. (2005). The global burden of diabetic foot disease. Lancet 366:1719–24. 16291066 10.1016/S0140-6736(05)67698-2

[CIT0013] Carobolante G, Mantaj J, Ferrari E, et al. (2020). Cow milk and intestinal epithelial cell-derived extracellular vesicles as systems for enhancing oral drug delivery. Pharmaceutics 12:226. 10.3390/pharmaceutics12030226 PMC7150822 32143503

[CIT0014] Chen Y, Gorski DH. (2008). Regulation of angiogenesis through a microRNA (miR-130a) that down-regulates antiangiogenic homeobox genes GAX and HOXA5. Blood 111:1217–26. 2008-02-01 17957028 10.1182/blood-2007-07-104133 PMC2214763

[CIT0015] Chen Y, Wang X. (2020). miRDB: an online database for prediction of functional microRNA targets. Nucleic Acids Res 48:D127–31. 31504780 10.1093/nar/gkz757 PMC6943051

[CIT0016] Chen J, Zhang Q, Liu D, et al. (2021). Exosomes: advances, development and potential therapeutic strategies in diabetic nephropathy. Metabolism 122:154834. 34217734 10.1016/j.metabol.2021.154834

[CIT0017] Cheung CC, Chung GT, Lun SW, et al. (2014). MiR-31 is consistently inactivated in EBV-associated nasopharyngeal carcinoma and contributes to its tumorigenesis. Mol Cancer 13:184. 25098679 10.1186/1476-4598-13-184 PMC4127521

[CIT0018] Chiba T, Cerqueira DM, Li Y, et al. (2021). Endothelial-derived miR-17 ∼ 92 promotes angiogenesis to protect against renal ischemia-reperfusion injury. J Am Soc Nephrol 32:553–62. 33514560 10.1681/ASN.2020050717 PMC7920169

[CIT0019] Chouhan D, Dey N, Bhardwaj N, et al. (2019). Emerging and innovative approaches for wound healing and skin regeneration: current status and advances. Biomaterials 216:119267. 31247480 10.1016/j.biomaterials.2019.119267

[CIT0020] Davis FM, Kimball A, Boniakowski A, et al. (2018). Dysfunctional wound healing in diabetic foot ulcers: new crossroads. Curr Diab Rep 18:2. 29362914 10.1007/s11892-018-0970-z

[CIT0021] Del Pozo-Acebo L, López De Las Hazas M, Tomé-Carneiro J, et al. (2021). Bovine milk-derived exosomes as a drug delivery vehicle for miRNA-based therapy. IJMS 22:1105. 33499350 10.3390/ijms22031105 PMC7865385

[CIT0022] EL Andaloussi S, Mäger I, Breakefield XO, et al. (2013). Extracellular vesicles: biology and emerging therapeutic opportunities. Nat Rev Drug Discov 12:347–57. 23584393 10.1038/nrd3978

[CIT0023] Faruqu FN, Xu L, Al-Jamal KT. (2018). Preparation of exosomes for siRNA delivery to cancer cells. J Vis Exp 142:10.3791/58814.. PMC6785346 30582600

[CIT0024] Feng L, Mumper RJ. (2013). A critical review of lipid-based nanoparticles for taxane delivery. Cancer Lett 334:157–75. 22796606 10.1016/j.canlet.2012.07.006 PMC3485436

[CIT0025] Grennan D. (2019). Diabetic foot ulcers. JAMA 321:114. 30620372 10.1001/jama.2018.18323

[CIT0026] Grossen P, Portmann M, Koller E, et al. (2021). Evaluation of bovine milk extracellular vesicles for the delivery of locked nucleic acid antisense oligonucleotides. Eur J Pharm Biopharm 158:198–210. 33248268 10.1016/j.ejpb.2020.11.012

[CIT0027] Ha D, Yang N, Nadithe V. (2016). Exosomes as therapeutic drug carriers and delivery vehicles across biological membranes: current perspectives and future challenges. Acta Pharm Sin B 6:287–96. 27471669 10.1016/j.apsb.2016.02.001 PMC4951582

[CIT0028] Herrmann IK, Wood MJA, Fuhrmann G. (2021). Extracellular vesicles as a next-generation drug delivery platform. Nat Nanotechnol 16:748–59. 34211166 10.1038/s41565-021-00931-2

[CIT0029] Hong WX, Hu MS, Esquivel M, et al. (2014). The role of hypoxia-inducible factor in wound healing. Adv Wound Care 3:390–9. 10.1089/wound.2013.0520 PMC4005494 24804159

[CIT0030] Hu J, Chen C, Liu Q, et al. (2015). The role of the miR-31/FIH1 pathway in TGF-β-induced liver fibrosis. Clin Sci 129:305–17. 10.1042/CS20140012 25728779

[CIT0031] Huang M, Nguyen P, Jia F, et al. (2011). Double knockdown of prolyl hydroxylase and factor-inhibiting hypoxia-inducible factor with nonviral minicircle gene therapy enhances stem cell mobilization and angiogenesis after myocardial infarction. Circulation 124:S46–S54. 21911818 10.1161/CIRCULATIONAHA.110.014019 PMC3181087

[CIT0032] Jeong K, Yu YJ, You JY, et al. (2020). Exosome-mediated microRNA-497 delivery for anti-cancer therapy in a microfluidic 3D lung cancer model. Lab Chip 20:548–57. 31942592 10.1039/c9lc00958b

[CIT0033] Jiao X, Sherman BT, Huang da W, et al. (2012). DAVID-WS: a stateful web service to facilitate gene/protein list analysis. Bioinformatics 28:1805–6. 22543366 10.1093/bioinformatics/bts251 PMC3381967

[CIT0034] Kandimalla R, Aqil F, Alhakeem SS, et al. (2021). Targeted oral delivery of paclitaxel using colostrum-derived exosomes. Cancers 13:3700. 34359601 10.3390/cancers13153700 PMC8345039

[CIT0035] Kandimalla R, Aqil F, Tyagi N, et al. (2021). Milk exosomes: a biogenic nanocarrier for small molecules and macromolecules to combat cancer. Am J Reprod Immunol 85:e13349. 32966664 10.1111/aji.13349

[CIT0036] Kim G, Kim M, Lee Y, et al. (2020). Systemic delivery of microRNA-21 antisense oligonucleotides to the brain using T7-peptide decorated exosomes. J Control Release 317:273–81. 31730913 10.1016/j.jconrel.2019.11.009

[CIT0037] Lando D, Peet DJ, Gorman JJ, et al. (2002). FIH-1 is an asparaginyl hydroxylase enzyme that regulates the transcriptional activity of hypoxia-inducible factor. Genes Dev 16:1466–71. 12080085 10.1101/gad.991402 PMC186346

[CIT0038] Li D, Li XI, Wang A, et al. (2015). MicroRNA-31 promotes skin wound healing by enhancing keratinocyte proliferation and migration. J Invest Dermatol 135:1676–85. 25685928 10.1038/jid.2015.48

[CIT0039] Li JH, Liu S, Zhou H, et al. (2014). starBase v2.0: decoding miRNA-ceRNA, miRNA-ncRNA and protein-RNA interaction networks from large-scale CLIP-Seq data. Nucleic Acids Res 42:D92–7. 24297251 10.1093/nar/gkt1248 PMC3964941

[CIT0040] Liang Y, Duan L, Lu J, et al. (2021). Engineering exosomes for targeted drug delivery. Theranostics 11:3183–95. 33537081 10.7150/thno.52570 PMC7847680

[CIT0041] Liang G, Kan S, Zhu Y, et al. (2018). Engineered exosome-mediated delivery of functionally active miR-26a and its enhanced suppression effect in HepG2 cells. Int J Nanomedicine 13:585–99. 29430178 10.2147/IJN.S154458 PMC5796471

[CIT0042] Liang G, Zhu Y, Ali DJ, et al. (2020). Engineered exosomes for targeted co-delivery of miR-21 inhibitor and chemotherapeutics to reverse drug resistance in colon cancer. J Nanobiotechnology 18:10. 31918721 10.1186/s12951-019-0563-2 PMC6950820

[CIT0043] Lim E, Jang E, Lee K, et al. (2013). Delivery of cancer therapeutics using nanotechnology. Pharmaceutics 5:294–317. 24300452 10.3390/pharmaceutics5020294 PMC3834952

[CIT0044] Liu Y, Ding M, Liu D, et al. (2015). MicroRNA profiling in cutaneous wounds of diabetic rats. Genet Mol Res 14:9614–25. 26345894 10.4238/2015.August.14.24

[CIT0045] Liu S, Sun Z, Zhou D, et al. (2017). Alkylated branched poly(β-amino esters) demonstrate strong DNA encapsulation, high nanoparticle stability and robust gene transfection efficacy. J Mater Chem B 5:5307–10. 32264068 10.1039/c7tb00996h

[CIT0046] Liu C, Tsai M, Hung P, et al. (2010). MiR-31 ablates expression of the HIF regulatory factor FIH to activate the HIF pathway in head and neck carcinoma. Cancer Res 70:1635–44. 20145132 10.1158/0008-5472.CAN-09-2291

[CIT0047] Luan X, Sansanaphongpricha K, Myers I, et al. (2017). Engineering exosomes as refined biological nanoplatforms for drug delivery. Acta Pharmacol Sin 38:754–63. 28392567 10.1038/aps.2017.12 PMC5520184

[CIT0048] Luo S, Sun X, Huang M, et al. (2021). Enhanced neuroprotective effects of epicatechin gallate encapsulated by bovine milk-derived exosomes against Parkinson's disease through antiapoptosis and antimitophagy. J Agric Food Chem 69:5134–43. 33890462 10.1021/acs.jafc.0c07658

[CIT0049] Ma T, Chen Y, Chen Y, et al. (2018). MicroRNA-132, delivered by mesenchymal stem cell-derived exosomes, promote angiogenesis in myocardial infarction. Stem Cells Int 2018:3290372. 30271437 10.1155/2018/3290372 PMC6151206

[CIT0050] Mahon PC, Hirota K, Semenza GL. (2001). FIH-1: a novel protein that interacts with HIF-1alpha and VHL to mediate repression of HIF-1 transcriptional activity. Genes Dev 15:2675–86. 11641274 10.1101/gad.924501 PMC312814

[CIT0051] Matsuda A, Moirangthem A, Angom RS, et al. (2020). Safety of bovine milk derived extracellular vesicles used for delivery of RNA therapeutics in zebrafish and mice. J Appl Toxicol 40:706–18. 31877238 10.1002/jat.3938

[CIT0052] Meng S, Cao J, Zhang X, et al. (2013). Downregulation of microRNA-130a contributes to endothelial progenitor cell dysfunction in diabetic patients via its target Runx3. PLoS One 8:e68611. 23874686 10.1371/journal.pone.0068611 PMC3709913

[CIT0053] Meng Z, Zhou D, Gao Y, et al. (2018). MiRNA delivery for skin wound healing. Adv Drug Deliv Rev 129:308–18. 29273517 10.1016/j.addr.2017.12.011

[CIT0054] Munagala R, Aqil F, Jeyabalan J, et al. (2016). Bovine milk-derived exosomes for drug delivery. Cancer Lett 371:48–61. 26604130 10.1016/j.canlet.2015.10.020 PMC4706492

[CIT0055] Munagala R, Aqil F, Jeyabalan J, et al. (2017). Exosomal formulation of anthocyanidins against multiple cancer types. Cancer Lett 393:94–102. 28202351 10.1016/j.canlet.2017.02.004 PMC5837866

[CIT0056] Nie X, Zhao J, Ling H, et al. (2020). Exploring microRNAs in diabetic chronic cutaneous ulcers: Regulatory mechanisms and therapeutic potential. Br J Pharmacol 177:4077–95. 32449793 10.1111/bph.15139 PMC7443474

[CIT0057] Ohtsuka M, Iwamoto K, Naito A, et al. (2021). Circulating microRNAs in gastrointestinal cancer. Cancers 13:3348. 34283058 10.3390/cancers13133348 PMC8267753

[CIT0058] Peng H, Kaplan N, Hamanaka RB, et al. (2012). MicroRNA-31/factor-inhibiting hypoxia-inducible factor 1 nexus regulates keratinocyte differentiation. Proc Natl Acad Sci USA 109:14030–4. 22891326 10.1073/pnas.1111292109 PMC3435188

[CIT0059] Petkovic M, Sørensen AE, Leal EC, et al. (2020). Mechanistic actions of microRNAs in diabetic wound healing. Cells 9:2228. 10.3390/cells9102228 PMC7601058 33023156

[CIT0060] Pirisinu M, Pham TC, Zhang DX, et al. (2020). Extracellular vesicles as natural therapeutic agents and innate drug delivery systems for cancer treatment: recent advances, current obstacles, and challenges for clinical translation. Semin Cancer Biol. 10.1016/j.semcancer.2020.08.007 32977006

[CIT0061] Plantz PE, Patton S, Keenan TW. (1973). Further evidence of plasma membrane material in skim milk. J Dairy Sci 56:978–83. 1973-01-01 4199911 10.3168/jds.S0022-0302(73)85292-0

[CIT0062] Rani S, Ritter T. (2016). The exosome – a naturally secreted nanoparticle and its application to wound healing. Adv Mater 28:5542–52. 26678528 10.1002/adma.201504009

[CIT0063] Rani P, Vashisht M, Golla N, et al. (2017). Milk miRNAs encapsulated in exosomes are stable to human digestion and permeable to intestinal barrier *in vitro*. J Funct Foods 34:431–9.

[CIT0064] Rawal S, Munasinghe PE, Shindikar A, et al. (2017). Down-regulation of proangiogenic microRNA-126 and microRNA-132 are early modulators of diabetic cardiac microangiopathy. Cardiovasc Res 113:90–101. 28065883 10.1093/cvr/cvw235

[CIT0065] Rey S, Semenza GL. (2010). Hypoxia-inducible factor-1-dependent mechanisms of vascularization and vascular remodelling. Cardiovasc Res 86:236–42. 20164116 10.1093/cvr/cvq045 PMC2856192

[CIT0066] Rodrigues M, Kosaric N, Bonham CA, et al. (2019). Wound healing: a cellular perspective. Physiol Rev 99:665–706. 30475656 10.1152/physrev.00067.2017 PMC6442927

[CIT0067] Rovira-Llopis S, Escribano-Lopez I, Diaz-Morales N, et al. (2018). Downregulation of miR-31 in diabetic nephropathy and its relationship with inflammation. Cell Physiol Biochem 50:1005–14. 30355913 10.1159/000494485

[CIT0068] Sadasivam M, Avci P, Gupta GK, et al. (2013). Self-assembled liposomal nanoparticles in photodynamic therapy. Eur J Nanomed 5(3):10.1515/ejnm-2013-0010. PMC3857307 24348377

[CIT0069] Sedykh S, Kuleshova A, Nevinsky G. (2020). Milk exosomes: perspective agents for anticancer drug delivery. IJMS 21:6646. 10.3390/ijms21186646 PMC7555228 32932782

[CIT0070] Sheng H, Hassanali S, Nugent C, et al. (2011). Insulinoma-released exosomes or microparticles are immunostimulatory and can activate autoreactive T cells spontaneously developed in nonobese diabetic mice. J Immunol 187:1591–600. 21734072 10.4049/jimmunol.1100231 PMC3150365

[CIT0071] Shi J, Ma X, Su Y, et al. (2018). MiR-31 mediates inflammatory signaling to promote re-epithelialization during skin wound healing. J Invest Dermatol 138:2253–63. 29605672 10.1016/j.jid.2018.03.1521 PMC6153075

[CIT0072] Smyth T, Kullberg M, Malik N, et al. (2015). Biodistribution and delivery efficiency of unmodified tumor-derived exosomes. J Control Release 199:145–55. 25523519 10.1016/j.jconrel.2014.12.013 PMC4441346

[CIT0073] Sorop A, Constantinescu D, Cojocaru F, et al. (2021). Exosomal microRNAs as biomarkers and therapeutic targets for hepatocellular carcinoma. Int J Mol Sci 22:4997. 34066780 10.3390/ijms22094997 PMC8125948

[CIT0074] Suárez Y, Wang C, Manes TD, et al. (2010). Cutting edge: TNF-induced microRNAs regulate TNF-induced expression of E-selectin and intercellular adhesion molecule-1 on human endothelial cells: feedback control of inflammation. J Immunol 184:21–5. 19949084 10.4049/jimmunol.0902369 PMC2797568

[CIT0075] Tang Y, Zhang Y, Chen Y, et al. (2015). The role of miR-19b in the inhibition of endothelial cell apoptosis and its relationship with coronary artery disease. Sci Rep 5:15132. 26459935 10.1038/srep15132 PMC4602285

[CIT0076] Tao S, Guo S, Li M, et al. (2017). Chitosan wound dressings incorporating exosomes derived from microRNA-126-overexpressing synovium mesenchymal stem cells provide sustained release of exosomes and heal full-thickness skin defects in a diabetic rat model. Stem Cells Transl Med 6:736–47. 28297576 10.5966/sctm.2016-0275 PMC5442792

[CIT0077] Tao H, Xu H, Zuo L, et al. (2020). Exosomes-coated bcl-2 siRNA inhibits the growth of digestive system tumors both *in vitro* and *in vivo*. Int J Biol Macromol 161:470–80. 32531356 10.1016/j.ijbiomac.2020.06.052

[CIT0078] Ullah M, Kodam SP, Mu Q, Akbar A. (2021). Microbubbles versus extracellular vesicles as therapeutic cargo for targeting drug delivery. ACS Nano 15:3612–20. 33666429 10.1021/acsnano.0c10689

[CIT0079] Umezu T, Tadokoro H, Azuma K, et al. (2014). Exosomal miR-135b shed from hypoxic multiple myeloma cells enhances angiogenesis by targeting factor-inhibiting HIF-1. Blood 124:3748–57. 25320245 10.1182/blood-2014-05-576116 PMC4263983

[CIT0080] Wang H, Huang T, Lo H, et al. (2014). Deficiency of the microRNA-31-microRNA-720 pathway in the plasma and endothelial progenitor cells from patients with coronary artery disease. Arterioscler Thromb Vasc Biol 34:857–69. 24558106 10.1161/ATVBAHA.113.303001

[CIT0081] Wang J, Tao J, Chen D, et al. (2014). MicroRNA miR-27b rescues bone marrow-derived angiogenic cell function and accelerates wound healing in type 2 diabetes mellitus. Arterioscler Thromb Vasc Biol 34:99–109. 24177325 10.1161/ATVBAHA.113.302104 PMC5533613

[CIT0082] Wong HA, Fatimy RE, Onodera C, et al. (2015). The cancer genome atlas analysis predicts microRNA for targeting cancer growth and vascularization in glioblastoma. Mol Ther 23:1234–47. 25903473 10.1038/mt.2015.72 PMC4817797

[CIT0083] Wu Y, Hu T, Chen Y, et al. (2011). The manipulation of miRNA-gene regulatory networks by KSHV induces endothelial cell motility. Blood 118:2896–905. 21715310 10.1182/blood-2011-01-330589

[CIT0084] Yang P, Cai L, Zhang G, et al. (2017). The role of the miR-17-92 cluster in neurogenesis and angiogenesis in the central nervous system of adults. J Neurosci Res 95:1574–81. 27869313 10.1002/jnr.23991

[CIT0085] Yang Y, Guo Z, Chen W, et al. (2021). M2 macrophage-derived exosomes promote angiogenesis and growth of pancreatic ductal adenocarcinoma by targeting E2F2. Mol Ther 29:1226–38. 33221435 10.1016/j.ymthe.2020.11.024 PMC7934635

[CIT0086] Yang F, Wang W, Zhou C, et al. (2015). MiR-221/222 promote human glioma cell invasion and angiogenesis by targeting TIMP2. Tumour Biol 36:3763–73. 25731730 10.1007/s13277-014-3017-3

[CIT0087] Yao X, Lyu P, Yoo K, et al. (2021). Engineered extracellular vesicles as versatile ribonucleoprotein delivery vehicles for efficient and safe CRISPR genome editing. J Extracell Vesicles 10:e12076. 33747370 10.1002/jev2.12076 PMC7962171

[CIT0088] Zeng Z, Li Y, Pan Y, et al. (2018). Cancer-derived exosomal miR-25-3p promotes pre-metastatic niche formation by inducing vascular permeability and angiogenesis. Nat Commun 9:5395. 30568162 10.1038/s41467-018-07810-w PMC6300604

[CIT0089] Zgheib C, Hilton SA, Dewberry LC, et al. (2019). Use of cerium oxide nanoparticles conjugated with microRNA-146a to correct the diabetic wound healing impairment. J Am Coll Surg 228:107–15. 30359833 10.1016/j.jamcollsurg.2018.09.017 PMC7846138

[CIT0090] Zhang J, Cai W, Fan Z, et al. (2019). MicroRNA-24 inhibits the oxidative stress induced by vascular injury by activating the Nrf2/Ho-1 signaling pathway. Atherosclerosis 290:9–18. 31539718 10.1016/j.atherosclerosis.2019.08.023

[CIT0091] Zhang Y, Sun X, Icli B, et al. (2017). Emerging roles for microRNAs in diabetic microvascular disease: novel targets for therapy. Endocr Rev 38:145–68. 28323921 10.1210/er.2016-1122 PMC5460677

[CIT0092] Zheng D, Huo M, Li B, et al. (2020). The role of exosomes and exosomal microRNA in cardiovascular disease. Front Cell Dev Biol 8:616161. 33511124 10.3389/fcell.2020.616161 PMC7835482

[CIT0093] Zhou D, Cutlar L, Gao Y, et al. (2016). The transition from linear to highly branched poly (β-amino ester)s: branching matters for gene delivery. Sci Adv 2:e1600102. 27386572 10.1126/sciadv.1600102 PMC4928911

[CIT0094] Zhu B, Cao X, Zhang W, et al. (2019). MicroRNA-31-5p enhances the Warburg effect via targeting FIH. FASEB J 33:545–56. 30004795 10.1096/fj.201800803R

[CIT0095] Zubair M, Ahmad J. (2019). Role of growth factors and cytokines in diabetic foot ulcer healing: a detailed review. Rev Endocr Metab Disord 20:207–17. 30937614 10.1007/s11154-019-09492-1

