# Supplementary figures and images for: Milk exosomes-mediated miR-31-5p delivery accelerates diabetic wound healing through promoting angiogenesis
Source: Drug Deliv. 2022 Jan 5;29(1):214–28. doi: 10.1080/10717544.2021.2023699 (PMC8741248; doi:10.1080/10717544.2021.2023699)

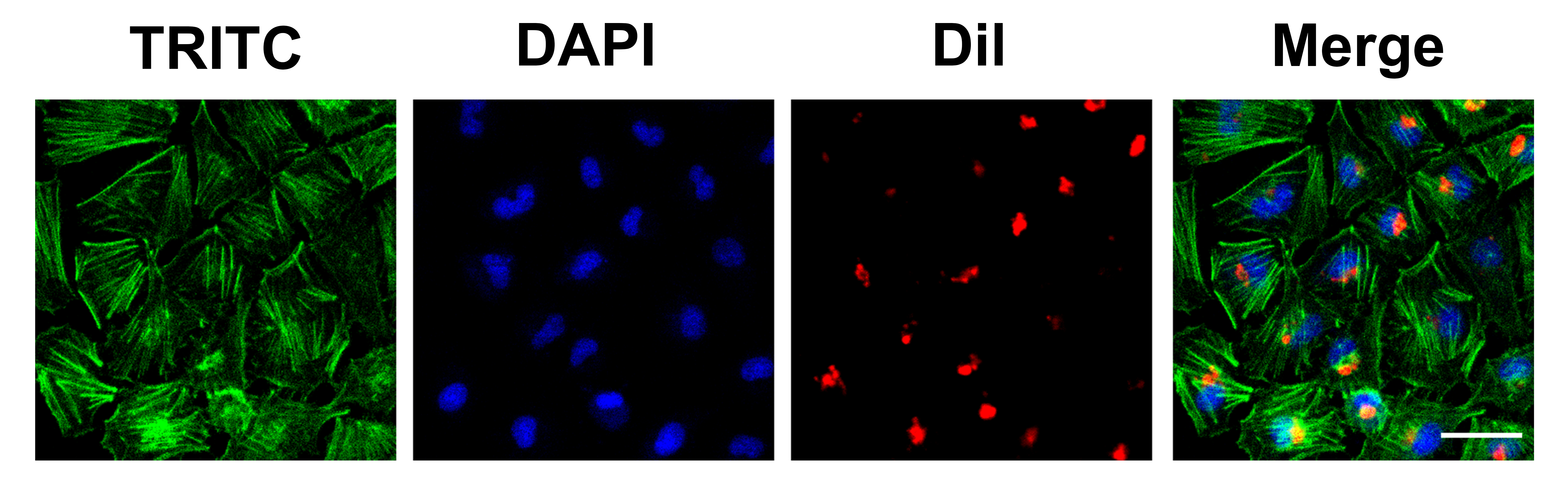

Supplement: Supplemental Material [file IDRD_A_2023699_SM8133.zip › FigSF2.jpg]

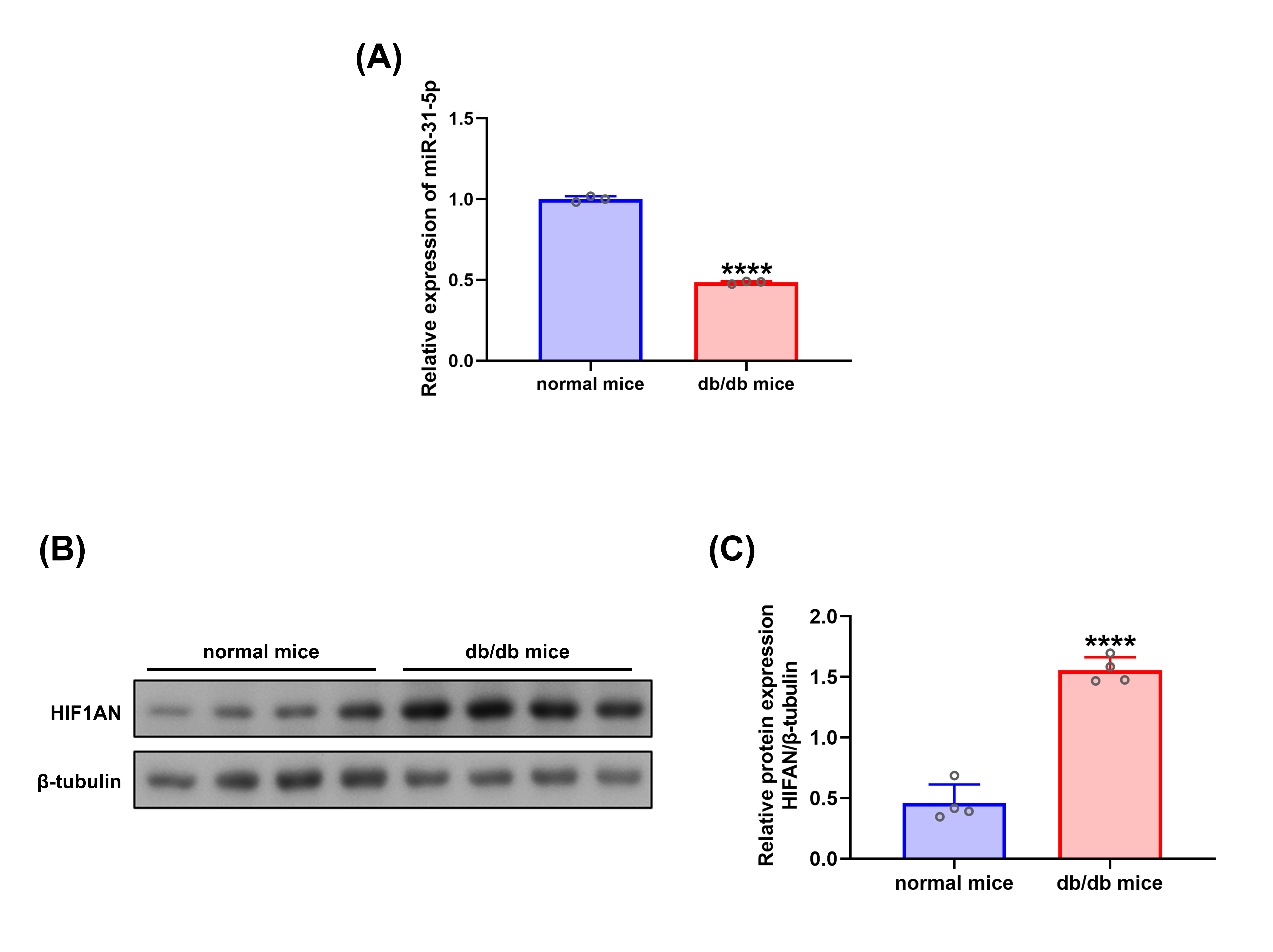

Supplement: Supplemental Material [file IDRD_A_2023699_SM8133.zip › SFig3.jpg]
